# Whole Genome Sequencing of the First H3N8 Equine Influenza Virus Identified in Malaysia

**DOI:** 10.3390/pathogens8020062

**Published:** 2019-05-10

**Authors:** Jacinta Gahan, Marie Garvey, Rozanah Asmah Abd Samad, Ann Cullinane

**Affiliations:** 1 Virology Unit, The Irish Equine Centre, Johnstown, Naas, Co. W91 RH93 Kildare, Ireland; JGahan@irishequinecentre.ie (J.G.); MGarvey@irishequinecentre.ie (M.G.); 2 Department of Veterinary Services, Federal Government Administration Centre, Putrajaya 62630, Malaysia; rozanah@dvs.gov.my

**Keywords:** genome sequencing, equine influenza, American lineage, Florida clade 1, Malaysia

## Abstract

In August 2015, Malaysia experienced an outbreak of acute respiratory disease in racehorses. Clinical signs observed were consistent with equine influenza (EI) infection. The index cases were horses recently imported from New Zealand. Rapid control measures, including temporary cancellation of racing, were implemented to minimize the impact of the outbreak. By November, the disease outbreak was resolved, and movement restrictions were lifted. The aim of this study was to confirm the clinical diagnosis and characterize the causal virus. A pan-reactive influenza type A real-time RT-PCR was used for confirmatory diagnosis. Antigenic characterization by haemagglutinin inhibition using a panel of specific ferret antisera indicated that the causal virus belonged to clade 1 of the H3N8 Florida sub-lineage. The genetic characterization was achieved by the whole genome sequencing of positive nasal swabs from clinically affected animals. Pylogenetic analysis of the haemagglutinin (HA) and neuraminidase (NA) genes demonstrated ≥99% homology with several EI strains that had recently circulated in the USA and Japan. The antigenic and genetic characterization did not indicate that the current World Organisation for Animal Health (OIE) recommendations for EI vaccine composition required modification.

## 1. Introduction

Equine influenza virus (EIV) can cause acute respiratory disease in horses, and lead to economically punitive disruptions within the equine industry [1,2,3]. EIV contains an octameric segmented genome encoding 12 proteins. Genotypic classification of EIV is traditionally based on the surface glycoproteins, haemagglutinin (HA) and neuraminidase (NA). Evolution of EIV typically occurs by mutations occurring within the antigenic epitopes of the strongly immunogenic HA protein, resulting in the evasion of the host immune response [4,5]. However, all 12 proteins are involved in EIV proliferation and host interaction, and should be taken into account to achieve a thorough analysis.

The H3N8 subtype of EIV has been in persistent circulation in endemic countries since the 1960s, and a gradual genomic evolution in the interim years has resulted in several antigenically distinct lineages. Following the equine influenza (EI) epizootics in the 1980s, H3N8 viruses diverged into the Eurasian and American lineages [6]. In the 1990s, the American lineage further drifted antigenically into the South America, Kentucky and Florida sub-lineages [7]. In the early 2000s, the Florida sub-lineage evolved into two distinct clades, known as Florida clades 1 and 2, which are currently the most epidemiologically relevant strains in circulation [5]. The Florida clade 1 and 2 (FC1, FC2) viruses have predominated in the US and Europe, respectively [1]. Additionally, both have caused sporadic outbreaks in diverse geographic locations, which highlights how the increased frequency of air travel has considerably reduced the time necessary for the spread of a virus between continents [8]. This is apparent from the introduction of the US-derived FC1 into South Africa [9], Japan [10], Australia [11] and Europe [5,12], and the European-derived FC2 into China [13], Mongolia [14] and India [15]. 

In EI endemic countries, EIV infection most commonly occurs as a result of an antigenic mismatch between the vaccine strain and the field strain, or by the existence of an unvaccinated or inadequately vaccinated population acting as an EIV reservoir. However, Malaysia is a country that has not previously experienced a disease outbreak caused by H3N8 EIV. In EI-free countries, the virus is typically introduced to susceptible populations by the international movement of infected horses that are not vaccinated with the most epidemiologically relevant EI strains to ensure protective cover [8,16]. Horses travel internationally more frequently than any other species, with the exception of humans, and therefore ensuring that all traveling animals are adequately vaccinated with the most antigenically relevant EI strains will help prevent this disease. To achieve this, phylogenetic and antigenic analyses of the circulating EI strains is undertaken annually by the OIE expert surveillance panel (ESP), and this information is used to inform/update the vaccine strain recommendations as necessary. The aims of this study were (i) to genetically and antigenically characterize the virus responsible for the first recorded outbreak of H3N8 EI in Malaysia, and (ii) to ascertain if the virus necessitated an update of OIE recommendations on the vaccine strain composition. 

## 2. Results

### 2.1. Disease Outbreak 

This EI outbreak commenced in August 2015. The index cases were horses that were recently imported into Malaysia from New Zealand via Singapore. The four horses arrived in Malaysia on the 31 July 2015. The horses were placed in a quarantine facility and vaccinated with the ProteqFlu (Merial) recombinant canary pox vaccine on the 13 August, two weeks following their arrival. On the 14 August one horse was moved to the Turf Club in Perak. The next day, the 15 August, two of the four horses were moved to Selangor Turf Club where they exhibited mild nasal discharge. On the 16 August the horse in Perak also had mild nasal discharge. On the 20 August the fourth horse was moved together with six other horses from Singapore, to Penang Turf Club. Upon arrival in Penang, one horse had nasal discharge. Nasal swabs were collected from ten horses at each of the turf clubs. Two horses in Selangor and two in Perak tested positive for EI on the 31 August. Disease control measures were implemented. The affected horses were quarantined, and temporary movement restrictions were imposed. Race meetings and other equestrian activities were suspended, as was the importation of horses. The samples collected between the 7 September and the 8 October from horses at the three turf clubs tested negative for EIV, although respiratory disease was reported at Selangor in late September. In November 2015, the outbreak was resolved, and the travel restrictions were lifted. 

### 2.2. Antigenic Characterisation

Antigenic characterization identified A/equine/Malaysia/1/2015 as a FC1 strain of EIV (Table 1). FC1 antisera had higher HI titres against A/equine/Malaysia/1/2015 than FC2 antisera. 

### 2.3. Phylogenetic Classification

**Figure 1:**
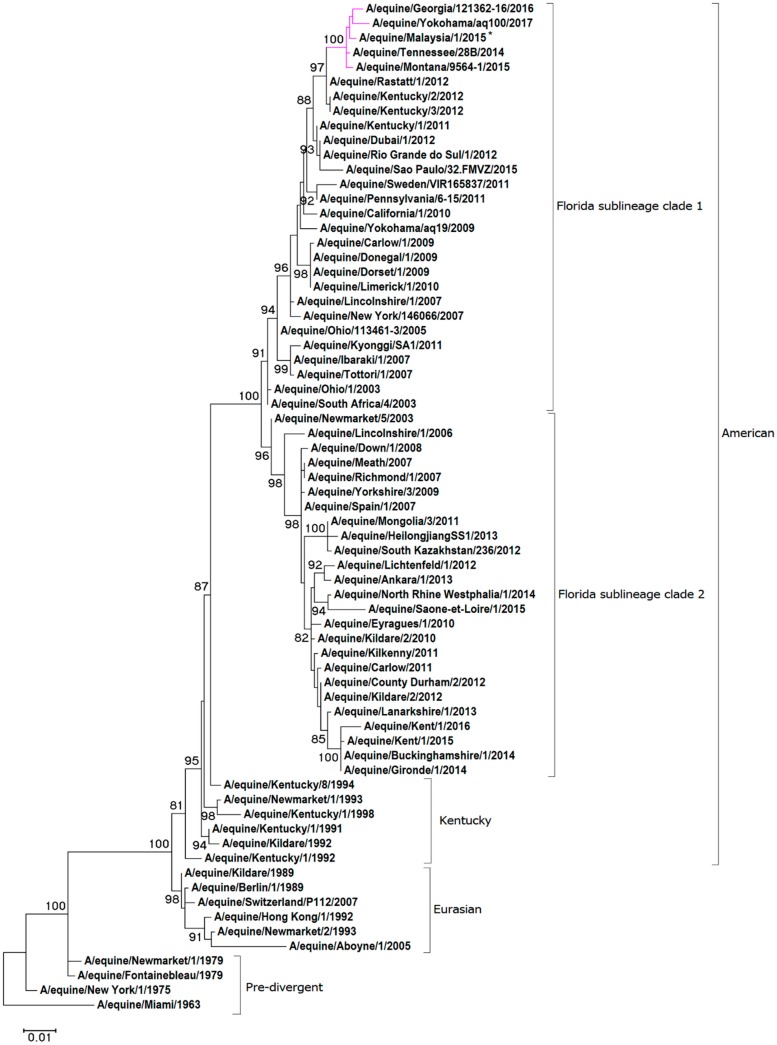
**Maximum likelihood tree of HA1 nucleotide sequences.** A phylogenetic tree of HA1 nucleotide sequences encoded by the H3N8 subtype of the EIV. Bootstrap values obtained after 1000 replicates are shown at major nodes.

**Figure 2:**
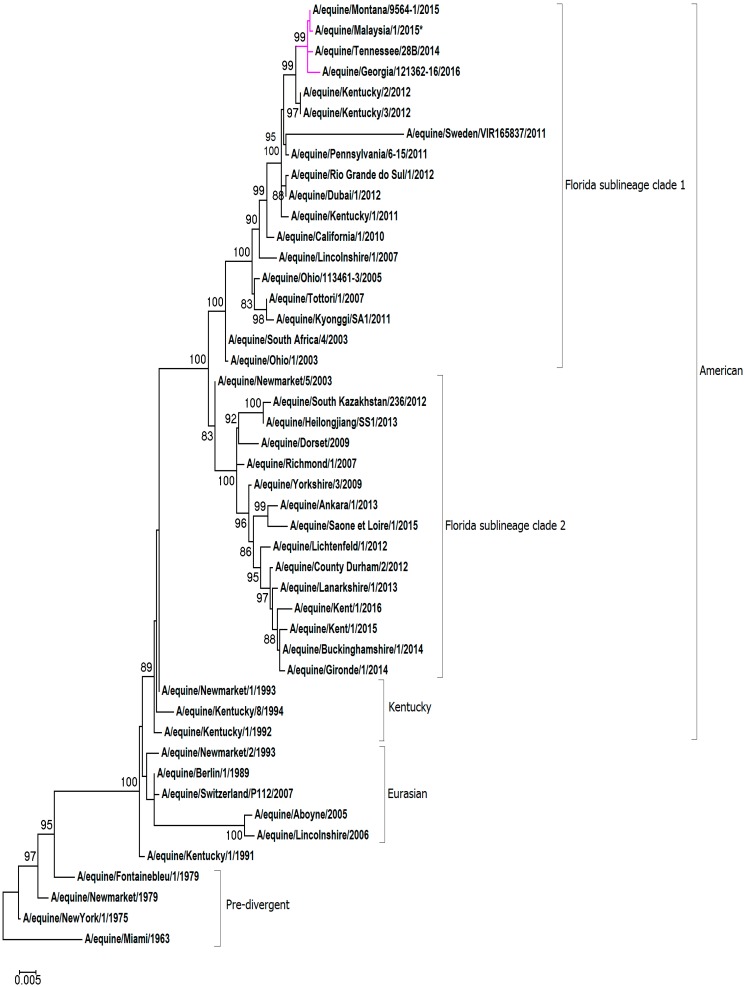
**Maximum likelihood tree of NA nucleotide sequences.** The phylogenetic tree of NA nucleotide sequences encoded by the H3N8 subtype of the EIV. Bootstrap values obtained after 1000 replicates are shown at major nodes.

For the phylogenetic analysis, representative HA1 and NA nucleotide sequences were obtained from pre-divergent, Eurasian and American lineages, as well as contemporary strains from the FC1 and FC2 sub-lineages. A maximum-likelihood phylogenetic tree based on HA1 gene encoding sequence confirmed that A/equine/Malaysia/1/2015 clusters with viruses of the FC1 lineage (see Figure 1). The HA1 sequence A/equine/Malaysia/1/2015 is closely related to FC1s that were recently isolated in the USA and Japan: A/equine/Tennessee/28B/2014, A/equine/Montana/9564-1/2015, A/equine/Georgia/121362-16/2016 and A/equine/Yokohama/aq100/2017. Analysis of the NA sequence demonstrated that A/equine/Malaysia/1/2015 is most closely related to the strains endemic in the USA in recent years: A/equine/Tennessee/28B/2014, A/equine/Montana/9564-1/2015, A/equine/Georgia/121362-16/2016 (see Figure 2). The NA sequence of A/equine/Yokohama/aq100/2017 was not available for comparison.

### 2.4. Whole Genome Sequencing 

Whole genome sequencing was achieved using overlapping M-13 labelled segment-specific primer pairs. The data generated have been deposited in GenBank with accession numbers MH135220.1 to MH135227.1. The predicted amino acid (aa) sequences of the assembled genome segments were aligned with A/equine/South Africa/4/2003 when possible, and with A/equine/Ohio/1/2003 for PB1 and PB1-F2 to ascertain changes in the aa sequences from the OIE-recommended FC1 vaccine strains (Supplementary Figures S1.1–S1.12). Within the RNA-dependent polymerase complex (PB2, PB1 and PA), a total of 11 (I63V, T377A, I398V, E578K, K660R, A661T, V667I, A684T, V686I, K699R, I754V), 8 (F94L, I114V, V200I, R584Q, K621R, V644I, V715A, R754G), and 9 (E59K, E237K, P259S, L348I, T354I, K385R, S409N, I465V, I505V) aa changes were observed, respectively. The PA-X gene is full length and has three (E59K, N237S, A240D) aa changes. The surface glycoproteins HA and NA have nine and eight aa changes, respectively (HA: S6N, G7D, S47P, R62K, D104N, A138S, N188T, V223I, D474N; NA: V35A, R252K, D258N, R260K, D266N, S337N, G416E, T434K). The non-structural proteins are PB1-F2 and NS1. The PB1-F2 segment is full length (90 aa) and not truncated to 81 aa as was observed in the FC1 isolates from 2003–2012. Amino acid positions 82–90 are identical to A/equine/Tennessee/28B/2014, A/equine/Montana/9564-1/2015 and A/equine/Georgia/121362-16/2016 (82W, 83Y, 84S, 85R, 86Q, 87E, 88W, 89T, 90N). The PB1-F2 segment has three (G50D, S63Y, R79Q) aa changes. NS1 has seven aa changes (V22F, E66K, T129I, I156V, H207N, N209I, P212S). The structural NP, M1 and NEP proteins have two (K214R, T430I), one (V15M) and one (M52L) aa changes, respectively. Finally, the M2 ion channel protein has one aa change (L59M). 

**Figure 3:**
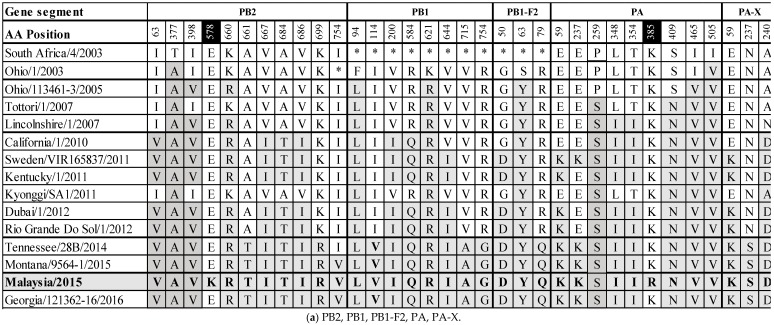

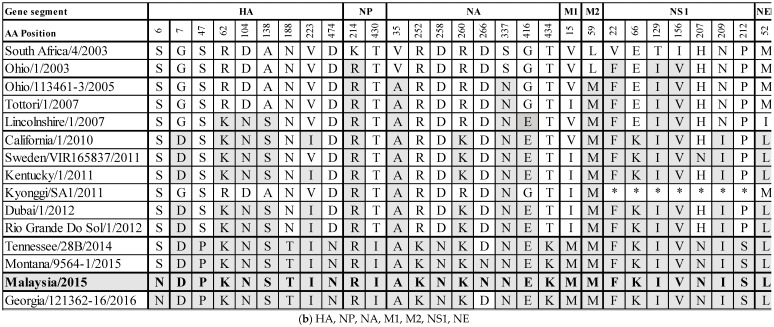
Amino acid differences between A/equine/South Africa/4/2003 or A/equine/Ohio/1/2003 and A/equine/Malaysia/1/2015, and a comparison to recent Florida clade 1 isolates (2003–2016).

To monitor the evolution of field viruses from the OIE-recommended FC1 vaccine strains, the substitutions from A/equine/SouthAfrica/4/2003 or A/equine/Ohio/1/2003 to A/equine/Malaysia/1/2015 were compared with other FC1 strains circulating globally in the interim years. The results are summarized in Figure 3a,b. The majority of substitutions occurring across all segments became highly conserved or fixed at certain points in time, for example, F94L and K621R in PB1, S63Y in PB1-F2, V35A and S337N in NA and L59M in M2 from 2005, P259S and S409N in PA from 2007, G7D in HA, R260K in NA and E66K and N209I in NS1 from 2010, M52L in NEP from 2010 and S47P, N188T and D474N in HA from 2014 based on the existing sequence information. Of the 63 substitutions across the EIV genome that exist from A/equine/South Africa/4/2003 or A/equine/Ohio/1/2003 to A/equine/Malaysia/1/2015, 28 (44%) occur within the polymerase complex proteins. Additionally, the only two unique-to-strain substitutions in A/equine/Malaysia/1/2015 are in PB2 (E578K) and PA (K385R). The immunogenic surface glycoproteins HA and NA contain 17 (27%) of the overall 63 substitutions, the majority of which became conserved at one of the above-mentioned four time points. The HA substitution S6N appears to be new and was not observed in the FC1 strains isolated before A/equine/Malaysia/1/2015, but it was subsequently observed in A/equine/Georgia/121362-16/2016. There are three substitutions in three different HA antigenic sites: R62K (site E), A138S (site A) and N188T (site B). The site E and site A substitutions emerged in 2007, and the site B substitution emerged in 2014. These three substitutions became fixed following their emergence. For NA, D266N has so far only been observed in A/equine/Malaysia/1/2015 and A/equine/Montana/9564-1/2015. Finally, the NS1 gene has seven substitutions which appear to have become highly conserved.

## 3. Discussion

The genome of EI viruses rely on a low-fidelity viral RNA polymerase complex without exonuclease proofreading capability for replication, and have an inherently high rate of error and substitution [17,18]. Individual or cumulative genetic changes occurring over time produce circulating viruses that are gradually antigenically drifting from an ancestral vaccine strain. Antibodies generated to closely related viruses are immunologically cross reactive [19]. However, in time, emerging viruses will drift to a point where they can evade the host humoral protection elicited by vaccination with the ancestral strain, and the vaccinated animals will no longer have optimal clinical or virological protection [6]. Global and temporal phylogenetic and antigenic surveillance of circulating EIV is paramount to ensuring that vaccines remain epidemiologically relevant. The aims of this study were to genetically and antigenically analyze the virus responsible for the first recorded outbreak of H3N8 EIV in Malaysia, and to ascertain if an update of the OIE recommendations on the vaccine strain composition was indicated. 

EIV was confirmed by RT-PCR as the etiological agent in this outbreak of respiratory disease in racehorses in Malaysia, in 2015. New Zealand, the country of origin of the index horses, is free of EI. A case of EI has never been recorded in New Zealand and vaccination is not practiced. As a result, the country is home to a large indigenous population of immunologically naïve horses which included the first horses to exhibit clinical signs in the Malaysian outbreak. At the time of the outbreak, Malaysia’s import regulations did not require that horses traveling from New Zealand be vaccinated against EI. The four horses imported from New Zealand had blood samples collected during a 14-day pre-export isolation period. Retrospective testing demonstrated that they were seronegative for EIV, and thus highly susceptible to infection. On arrival in Malaysia, the horses were quarantined, and vaccination for EI was provided on day 14 post arrival, one to five days before the horses left the quarantine facility. Clinical signs were first observed in two of the horses on the same day that they were moved to Selangor Turf Club, and a day later in a third horse, within 48 hours of arrival at the Perak Turf Club (i.e., before the horses had responded to the vaccination). The source of virus to the New Zealand horses was not identified but may have been infected vaccinated horses in the quarantine facility. In a study on equine disease events occurring as a result of international horse movement, EI was identified as the most frequent cause of disease events due to partially vaccinated and/or un-vaccinated animals, inconsistent isolation of newly introduced horses and lapses in bio-security protocols [20]. In recent years, FC1 strains of EIV have caused outbreaks in Japan and Australia in 2007 [2,10,21], South America with an incursion to Dubai in 2012 [3,22], Brazil and the USA in 2015 [23,24], and Pakistan in 2016 [25]. Key strategies to avoid such disease outbreaks include the availability of the most epidemiologically relevant vaccine strains via global surveillance and virus characterization. 

A maximum likelihood phylogenetic tree generated for the HA1 and NA of A/equine/Malaysia/1/2015 revealed that the strain clustered closely with recent isolates of the FC1 sub-lineage from the USA and Japan. The substitution rate for HA and NA was high between A/equine/Malaysia/1/2015 and the vaccine strain, and three of the HA1 substitutions were in known antigenic sites. Despite this, antigenic analysis with clade-specific ferret antisera did not highlight an evident difference in antigenicity, and A/equine/Malaysia/1/2015 was found to be strongly cross-reactive with all FC1 strains that were investigated. The virus characteristics did not indicate any necessity to modify the current OIE recommendations for EI vaccine composition. However, the accumulation of aa changes in the immunogenic HA1 and NA of A/equine/Malaysia/1/2015 is a timely reminder that the virus continues to evolve, and that the protection conferred by the vaccine strain needs to be closely monitored. Early detection of antigenic drift is pivotal to the maintenance of immunologically relevant vaccines [22,26,27]. 

The traditional HA1- and NA-centric approaches to studying EIV evolution have contributed enormously to the goal of understanding the epidemiology of EI. However, the whole genome sequencing of the Malaysian virus was undertaken in this study to monitor the EIV strain circulation, enrich the bank of available EIV sequence data and to facilitate future genome-based studies of influenza virulence. The genome of A/equine/Malaysia/1/2015 was analyzed and the possibility of reassortment was eliminated for all the eight segments and 12 encoded proteins. The sites of substitution between A/equine/Malaysia/1/2015 and the OIE-recommended FC1 vaccine strain A/equine/South Africa/4/2003 (A/equine/Ohio/1/2003 for PB1 and PB1-F2) were examined for a comparison of the available contemporary circulating FC1 strains from 2003 to 2016. This comparison revealed that numerous cross-genome substitutions for FC1 strains had occurred simultaneously at particular points in time and thereafter became fixed. An analogous pattern was observed in FC2 viruses in Europe from 2007–2015 [28]. Whole genome sequencing of a FC2 virus responsible for the first recorded outbreak in Turkey in 2013 revealed that 37 substitutions had emerged across the EI genome at different points in time, many of which became fixed since the recommended vaccine strain was isolated in 2007 [28]. In the 2015 Malaysian FC1 outbreak, 63 substitutions were observed when compared with the vaccine strains isolated in 2003. The polymerase complex responsible for viral replication contains 43% of these aa substitutions, and only two unique- to -strain substitutions, which are in the PB2 (E578K) and PA (K385R) segments. Although the possible effect of the accumulation of such mutations on viral replication, virulence and immune evasion merits investigation, the prompt resolution of the outbreak in Malaysia suggests that this virus strain was not particularly virulent. In fact, the clinical signs reported in the New Zealand horses were very mild, given that they did not have adequate time to mount an effective immune response to the vaccination, and thus were very susceptible.

There are over 4000 horses in Malaysia, but it appears that virus spread was successfully curtailed by the temporary cancellation of horse raceing and the export/import of horses. The Malayan Racing Association requires that all horses at the turf clubs receive a primary course of two EI vaccines, four to six weeks apart, followed by booster vaccinations at six-monthly intervals. It appears that this compulsory vaccination program limited the effects of the virus incursion. The ban on horse movement at the turf clubs was maintained for three weeks after the clinical signs had disappeared. The outbreak was resolved three months after the identification of the index cases, and restrictions were lifted. Such restrictions are costly, and the Malaysian authorities have put a protocol in place to minimize the risk in future. The current regulation requires that, for importation of horses from New Zealand to Malaysia, a veterinary certificate is provided confirming that the animal has been vaccinated against equine influenza with a primary course (vaccinated twice), or a booster between 21 and 90 days before shipment.

Finally, while a high aa substitution rate was observed when A/equine/Malaysia/1/2015 was compared to the recommended clade 1 vaccine strains, there was no antigenic evidence necessitating modification of the OIE recommendations for vaccine strain composition. 

## 4. Materials and Methods 

### 4.1. Clinical Detection

Nasopharyngeal swabs were collected from four clinically affected horses in Malaysia in August 2015. A pan-reactive influenza type A real time RT-PCR described by Heine et al 2007 was used to confirm EIV as the etiologic agent [29]. 

### 4.2. Virus Isolation

Virus isolation of one EI RT-PCR positive nasopharyngeal swab was carried out in 10-day-old embryonated hens’ eggs [12]. A/equine/Malaysia/1/2015 at passage 2 (P2) had a HA titre of 1:64, which was sufficient for subsequent antigenic characterization.

### 4.3. Antigenic Characterization

A/equine/Malaysia/1/2015 was antigenically characterized by the haemagglutination inhibition (HI) test with clade-specific ferret antisera. In summary, four HA units of each virus were tested with serial dilutions of ferret antisera against A/equine/Newmarket/1/1993 and A/equine/Newmarket/2/1993 (representative strains of American and Eurasian lineages obtained from the OIE Reference Laboratory, Animal Health Trust, Newmarket, UK); A/equine/South-Africa/4/2003 and A/equine/Donegal/2009 (representative strains of the FC1 sub-lineage); and A/equine/Meath/2007 and A/equine/Kildare/2012 (representative strains of the FC2 sub-lineage). 

### 4.4. Whole Genome Sequencing 

RNA was extracted directly from nasopharyngeal swab secretions using QIAamp Viral RNA Mini Kit (Qiagen, as per manufacturer’s instructions). For whole genome sequencing (WGS), one-step PCR was undertaken using SuperScript™ III One-Step RT-PCR System with Platinum® Taq High Fidelity (Invitrogen, per manufacturer’s instructions) using overlapping M-13 labelled primers for each of the eight EIV segments [30]. Thermocycling conditions were as follows: reverse transcription at 55 °C (30 min) and initial denaturation at 94 °C (2 min), 40 cycles of denaturation at 94 °C (60 s), annealing gradient of 45–60 °C (10 s), elongation at 60 °C (60 s) and final elongation at 60 °C for 5 min. PCR products were visualized on a 1% agarose gel stained with 0.0003% Sybersafe (Invitrogen). PCR amplicons were purified using a QIAquick PCR purification kit (Qiagen, as per manufacturer’s instructions) and sequenced (GATC, Germany).

The genome segments were assembled using DNASTAR SeqMan^TM^ II (Madison, WI). Multiple nucleotide and amino acid sequence alignments of each segment were constructed using the ClustalW [31] accessory application in the BioEdit sequence alignment editor version 7.0.9.0 [32]. Maximum likelihood trees were constructed using Mega 7 version 7.0.21 [33] with HA1 and NA sequences mined from NCBI GenBank and GISAID databases [34]. The lowest Baysian information criterion score dictated the optimum model chosen for each tree. Following the phylogenetic classification as FC1 based on the HA1 gene, the full-genome sequence of A/equine/Malaysia/1/2015 was aligned with the OIE-recommended FC1 representative vaccine strains A/equine/South Africa/4/2003 and A/equine/Ohio/1/2003 as reference. Amino acid changes identified were then compared to other recently circulating FC1 strains.

## Figures and Tables

**Table 1:** Antigenic characterization of A/equine/Malaysia/1/2015 using ferret antisera.

	Antisera (Geometric Mean Titre)					
	NM/1/93	NM/2/93	SA/4/03	Donegal/09	Meath/07	Kildare/12
**Antigen**	Am	Eur	FC1	FC1	FC2	FC2
NM/1/93	**256**	64	256	256	512	1024
NM/2/93	128	**256**	32	32	256	256
SA/04/03	51	25	**813**	1024	323	512
Donegal/09	161	81	813	**1625**	203	203
Meath/07	203	102	81	81	**406**	323
Kildare/12	203	102	161	128	1024	**1290**
*Malaysia/1/2015*	64	16	512	645	64	102

American lineage (Am), European lineage (Eur), Florida clades 1 and 2 of the American lineage (FC1 and FC2). NM/1/93 = A/equine/Newmarket/1/93, NM/2/93 = A/equine/Newmarket/2/93, SA/4/03 = A/equine/South Africa/4/03, Donegal/09 = A/equine/Donegal/09, Meath/07 = A/equine/Meath/07 and Kildare/12 = A/equine/Kildare/12. Homologous titres are in bold. Highest titre to recent clade 1 antisera is shaded. Solid box = clade 1 antisera titres against clade 1 viruses, dotted box = clade 2 antisera titres against clade 2 viruses.

## Supplementary Materials

The following are available online at https://www.mdpi.com/2076-0817/8/2/62/s1. Supplementary Figures S1.1–S1.12: Amino acid alignments of the predicted protein sequences of A/equine/Malaysia/1/2015 and selected EIV Florida sub-lineage clade 1 strains against OIE reference strain A/equine/South Africa/4/2003 and A/equine/Ohio/1/2003. Table S1: Accession codes for EIV HA1 and NA sequences included in phylogenetic analysis Figure 1 and Figure 2, respectively. Table S2: Accession codes for sequences used in Figure 3 and Supplementary Figures S1.1–S1.12.
