# Supplementary material for: Whole Genome Sequencing of the First H3N8 Equine Influenza Virus Identified in Malaysia
Source: Pathogens. 2019 May 10;8(2):62. doi: 10.3390/pathogens8020062 (PMC6630255; doi:10.3390/pathogens8020062)
Supplement: Supplementary file 1 [file pathogens-08-00062-s001.zip › SupplementaryFiles/Supplementary Figures S1.1-S1.12.docx]

**Supplementary figures S1.1- 1.12**

# Amino acid alignment of the predicted protein sequences of A/equine/Malaysia/2015 and selected EIV Florida sub-lineage clade 1 strains against OIE reference strain A/equine/South Africa/4/2003 and A/equine/Ohio/1/2003. Amino acid identity is represented with a dot. Dashes indicate sequence at this position was unavailable.

# **Figure S1.1 PB2 Alignment**

***10 20 30 40 50 60 70 80 90 100***

***....|....| ....|....| ....|....| ....|....| ....|....| ....|....| ....|....| ....|....| ....|....| ....|....|***

**South Africa/4/2003**  **MERIKELRDL** **MLQSRTREIL** **TKTTVDHMAI** **IKKYTSGRQE** **KNPALRMKWM** **MAMKYPITAD** **KRIMEMIPER** **NEQGQTLWSK** **TNDAGSDRVM** **VSPLAVTWWN**

**Ohio/1/2003**  **----------** **-.........** **..........** **..........** **..........** **..........** **..........** **..........** **..........** **..........**

**Ohio/113461-3/2005**  **..........** **..........** **..........** **..........** **..........** **..........** **..........** **..........** **..........** **..........**

**Tottori/1/2007**  **----------** **..........** **..........** **..........** **..........** **..........** **..........** **........N.** **..........** **..........**

**Lincolnshire/1/2007**  **..........** **..........** **..........** **..........** **..........** **..........** **..........** **..........** **..........** **..........**

**California/1/2010**  **..........** **..........** **..........** **..........** **..........** **..........** **..V.......** **..........** **M.........** **..........**

**Sweden/VIR165837/2011**  **..........** **..........** **..........** **..........** **..........** **..........** **..V.......** **..........** **..........** **..........**

**Kentucky/1/2011**  **..........** **..........** **..........** **..........** **..........** **..........** **..V.......** **..........** **..........** **..........**

**Kyonggi/SA1/2011**  **..........** **..........** **..........** **..........** **..........** **..........** **..........** **........N.** **..........** **..........**

**Dubai/1/2012**  **..........** **..........** **..........** **..........** **..........** **..........** **..V..V....** **..........** **..........** **..........**

**Rio Grande do Sul/1/2012** **..........** **..........** **..........** **..........** **..........** **..........** **..V.......** **..........** **..........** **..........**

**Tennessee/28B/2014**  **..........** **..........** **..........** **..........** **..........** **..........** **..V.......** **..........** **..........** **..........**

**Montana/9564-1/2015**  **..........** **..........** **..........** **..........** **..........** **..........** **..V.......** **..........** **..........** **..........**

**Malaysia/1/2015**  **..........** **..........** **..........** **..........** **..........** **..........** **..V.......** **..........** **..........** **..........**

**Georgia/121362-16/2016**  **..........** **..........** **..........** **..........** **..........** **..........** **..V.K.....** **..........** **..........** **..........**

***110 120 130 140 150 160 170 180 190 200***

***....|....| ....|....| ....|....| ....|....| ....|....| ....|....| ....|....| ....|....| ....|....| ....|....|***

**South Africa/4/2003**  **RNGPTTSTIH** **YPKVYKTYFE** **KVERLKHGTF** **GPVHFRNQVK** **IRRRVDVNPG** **HADLSAKEAQ** **DVIMEVVFPN** **EVGARILTSE** **SQLTITKEKK** **EELQDCKIAP**

**Ohio/1/2003**  **..........** **..........** **..........** **..........** **..........** **..........** **..........** **..........** **..........** **..........**

**Ohio/113461-3/2005**  **..........** **..........** **..........** **..........** **..........** **..........** **..........** **..........** **..........** **..........**

**Tottori/1/2007**  **..........** **..........** **..........** **..........** **..........** **..........** **..........** **..........** **..........** **..........**

**Lincolnshire/1/2007**  **..........** **..........** **..........** **..........** **..........** **..........** **..........** **..........** **..........** **..........**

**California/1/2010**  **..........** **..........** **..........** **..........** **..........** **..........** **..........** **..........** **..........** **..........**

**Sweden/VIR165837/2011**  **..........** **..........** **..........** **..........** **..........** **..........** **..........** **..........** **..........** **..........**

**Kentucky/1/2011**  **..........** **..........** **..........** **..........** **..........** **..........** **..........** **..........** **..........** **..........**

**Kyonggi/SA1/2011**  **..........** **..........** **..........** **..........** **..........** **..........** **..........** **..........** **..........** **..........**

**Dubai/1/2012**  **..........** **..........** **..........** **..........** **..........** **..........** **..........** **..........** **..........** **..........**

**Rio Grande do Sul/1/2012** **..........** **..........** **..........** **..........** **..........** **..........** **..........** **..........** **..........** **..........**

**Tennessee/28B/2014**  **..........** **..........** **..........** **..........** **..........** **..........** **..........** **..........** **..........** **..........**

**Montana/9564-1/2015**  **..........** **..........** **..........** **..........** **..........** **..........** **..........** **..........** **..........** **..........**

**Malaysia/1/2015**  **..........** **..........** **..........** **..........** **..........** **..........** **..........** **..........** **..........** **..........**

**Georgia/121362-16/2016**  **..........** **..........** **..........** **..........** **..........** **..........** **..........** **..........** **..........** **..........**

***210 220 230 240 250 260 270 280 290 300***

***....|....| ....|....| ....|....| ....|....| ....|....| ....|....| ....|....| ....|....| ....|....| ....|....|***

**South Africa/4/2003**  **LMVAYMLERE** **LVRKTRFLPV** **AGGTSSVYIE** **VLHLTQGTCW** **EQMYTPGGEV** **RNDDIDQSLI** **IAARNIVRRA** **TVSADPLASL** **LEMCHSTQIG** **GIRMVDILKQ**

**Ohio/1/2003**  **..........** **.I........** **..........** **..........** **..........** **..........** **..........** **..........** **..........** **..........**

**Ohio/113461-3/2005**  **..........** **..........** **..........** **..........** **..........** **..........** **..........** **..........** **..........** **..........**

**Tottori/1/2007**  **..........** **..........** **..........** **..........** **..........** **..........** **..........** **..........** **..........** **.........H**

**Lincolnshire/1/2007**  **..........** **..........** **..........** **..........** **..........** **..........** **..........** **..........** **..........** **..........**

**California/1/2010**  **..........** **..........** **..........** **..........** **..........** **..........** **..........** **..........** **..........** **..........**

**Sweden/VIR165837/2011**  **..........** **..........** **..........** **..........** **..........** **..........** **..........** **..........** **..........** **..........**

**Kentucky/1/2011**  **..........** **..........** **..........** **..........** **..........** **..........** **..........** **..........** **..........** **..........**

**Kyonggi/SA1/2011**  **..........** **..........** **..........** **..........** **..........** **..........** **..........** **..........** **..........** **.........H**

**Dubai/1/2012**  **..........** **..........** **..........** **..........** **..........** **..........** **..........** **..........** **..........** **..........**

**Rio Grande do Sul/1/2012** **..........** **..........** **..........** **..........** **..........** **..........** **..........** **..........** **..........** **..........**

**Tennessee/28B/2014**  **..........** **..........** **..........** **..........** **..........** **..........** **..........** **..........** **..........** **..........**

**Montana/9564-1/2015**  **..........** **..........** **..........** **..........** **..........** **..........** **..........** **..........** **..........** **..........**

**Malaysia/1/2015**  **..........** **..........** **..........** **..........** **..........** **..........** **..........** **..........** **..........** **..........**

**Georgia/121362-16/2016**  **..........** **..........** **..........** **..........** **..........** **..........** **..........** **..........** **..........** **..........**

***310 320 330 340 350 360 370 380 390 400***

***....|....| ....|....| ....|....| ....|....| ....|....| ....|....| ....|....| ....|....| ....|....| ....|....|***

**South Africa/4/2003**  **NPTEEQAVDI** **CKAAMGLRIS** **SSFSFGGFTF** **KRTSGSSVKR** **EEEMLTGNLQ** **TLKIRVHEGY** **EEFTMVGRRA** **TAILRKTTRR** **LIQLIVSGRD** **EQSIAEAIIV**

**Ohio/1/2003**  **..........** **..........** **..........** **..........** **..........** **..........** **..........** **......A...** **..........** **..........**

**Ohio/113461-3/2005**  **..........** **..........** **..........** **..........** **..........** **..........** **..........** **......A...** **..........** **.......V..**

**Tottori/1/2007**  **..........** **..........** **..........** **..........** **..........** **..........** **..........** **......A...** **..........** **..........**

**Lincolnshire/1/2007**  **..........** **..........** **..........** **..........** **..........** **..........** **..........** **......A...** **..........** **.......V..**

**California/1/2010**  **..........** **..........** **..........** **..........** **......S...** **..........** **..........** **......A...** **..........** **.......V..**

**Sweden/VIR165837/2011**  **..........** **..........** **..........** **..........** **..........** **..........** **..........** **......A...** **..........** **.......V..**

**Kentucky/1/2011**  **..........** **..........** **..........** **..........** **..........** **..........** **..........** **......A...** **..........** **.......V..**

**Kyonggi/SA1/2011**  **..........** **..........** **..........** **..........** **..........** **..........** **..........** **......A...** **..........** **..........**

**Dubai/1/2012**  **..........** **..........** **..........** **..........** **..........** **..........** **..........** **......A...** **..........** **.......V..**

**Rio Grande do Sul/1/2012** **..........** **..........** **..........** **..........** **..........** **..........** **..........** **......A...** **..........** **.......V..**

**Tennessee/28B/2014**  **..........** **..........** **..........** **..........** **..........** **..........** **..........** **....K.A...** **..........** **.......V..**

**Montana/9564-1/2015**  **..........** **..........** **..........** **..........** **..........** **..........** **..........** **......A...** **..........** **.......V..**

**Malaysia/1/2015**  **..........** **..........** **..........** **..........** **..........** **..........** **..........** **......A...** **..........** **.......V..**

**Georgia/121362-16/2016**  **..........** **..........** **..........** **..........** **..........** **..........** **..........** **......A...** **..........** **.......V..**

***410 420 430 440 450 460 470 480 490 500***

***....|....| ....|....| ....|....| ....|....| ....|....| ....|....| ....|....| ....|....| ....|....| ....|....|***

**South Africa/4/2003**  **AMVFSQEDCM** **IKAVRGDLNF** **VNRANQRLNP** **MHQLLRHFQK** **DAKVLFQNWG** **IEPIDNVMGM** **IGILPDMTPS** **TEMSLRGVRV** **SKMGVDEYSS** **TERVVVSIDR**

**Ohio/1/2003**  **..........** **..........** **..........** **..........** **..........** **..........** **..........** **..........** **..........** **..........**

**Ohio/113461-3/2005**  **..........** **V.........** **..........** **..........** **..........** **..........** **..........** **..........** **..........** **..........**

**Tottori/1/2007**  **..........** **..........** **..........** **..........** **..........** **..........** **..........** **..........** **..........** **..........**

**Lincolnshire/1/2007**  **..........** **..........** **..........** **..........** **..........** **..........** **..........** **..........** **..........** **..........**

**California/1/2010**  **..........** **..........** **..........** **..........** **..........** **..........** **..........** **..........** **..........** **..........**

**Sweden/VIR165837/2011**  **..........** **..........** **..........** **..........** **..........** **..........** **..........** **..........** **..........** **..........**

**Kentucky/1/2011**  **..........** **..........** **..........** **..........** **..........** **..........** **..........** **..........** **..........** **..........**

**Kyonggi/SA1/2011**  **..........** **..........** **..........** **..........** **..........** **..........** **..........** **..........** **..........** **..........**

**Dubai/1/2012**  **..........** **..........** **..........** **..........** **..........** **..........** **..........** **..........** **..........** **..........**

**Rio Grande do Sul/1/2012** **..........** **..........** **..........** **..........** **..........** **..........** **..........** **..........** **..........** **..........**

**Tennessee/28B/2014**  **..........** **..........** **..........** **..........** **..........** **..........** **..........** **..........** **..........** **..........**

**Montana/9564-1/2015**  **..........** **..........** **..........** **..........** **..........** **..........** **..........** **..........** **..........** **..........**

**Malaysia/1/2015**  **..........** **..........** **..........** **..........** **..........** **..........** **..........** **..........** **..........** **..........**

**Georgia/121362-16/2016**  **..........** **..........** **..........** **..........** **..........** **..........** **..........** **..........** **..........** **..........**

***510 520 530 540 550 560 570 580 590 600***

***....|....| ....|....| ....|....| ....|....| ....|....| ....|....| ....|....| ....|....| ....|....| ....|....|***

**South Africa/4/2003**  **FLRVRDQRGN** **ILLSPEEVSE** **TQGTEKLTII** **YSSSMMWEIN** **GPESVLVNTY** **QWIIRNWEIV** **KIQWSQDPTM** **LYNKIEFEPF** **QSLVPRATRS** **QYSGFVRTLF**

**Ohio/1/2003**  **..........** **..........** **..........** **..........** **..........** **..........** **..........** **..........** **..........** **..........**

**Ohio/113461-3/2005**  **..........** **..........** **..........** **..........** **..........** **..........** **..........** **..........** **..........** **..........**

**Tottori/1/2007**  **..........** **..........** **..........** **..........** **..........** **..........** **.........I** **...R......** **..........** **..........**

**Lincolnshire/1/2007**  **..........** **..........** **..........** **..........** **..........** **..........** **..........** **..........** **..........** **..........**

**California/1/2010**  **..........** **..........** **..........** **..........** **..........** **.........I** **..........** **..........** **..........** **..........**

**Sweden/VIR165837/2011**  **..........** **..........** **..........** **..........** **..........** **..........** **..........** **..........** **..........** **..........**

**Kentucky/1/2011**  **..........** **..........** **..........** **..........** **..........** **..........** **..........** **..........** **..........** **..........**

**Kyonggi/SA1/2011**  **..........** **..........** **..........** **..........** **..........** **..........** **.........I** **...R......** **..........** **.......I..**

**Dubai/1/2012**  **..........** **..........** **..........** **..........** **..........** **..........** **..........** **..........** **..........** **..........**

**Rio Grande do Sul/1/2012** **..........** **..........** **..........** **..........** **..........** **..........** **..........** **..........** **..........** **..........**

**Tennessee/28B/2014**  **..........** **..........** **..........** **..........** **..........** **..........** **..........** **..........** **..........** **..........**

**Montana/9564-1/2015**  **..........** **..........** **..........** **..........** **..........** **..........** **..........** **..........** **..........** **..........**

**Malaysia/1/2015**  **..........** **..........** **..........** **..........** **..........** **..........** **..........** **.......K..** **..........** **..........**

**Georgia/121362-16/2016**  **..........** **..........** **..........** **..........** **..........** **..........** **..........** **..........** **..........** **..........**

***610 620 630 640 650 660 670 680 690 700***

***....|....| ....|....| ....|....| ....|....| ....|....| ....|....| ....|....| ....|....| ....|....| ....|....|***

**South Africa/4/2003**  **QQMRDVLGTF** **DTAQIIKLLP** **FAAAPPEQSR** **MQFSSLTVNV** **RGSGMRILVR** **GNSPVFNYNK** **ATKRLTVLGK** **DAGALTEDPD** **EGTAGVESAV** **LRGFLILGKE**

**Ohio/1/2003**  **..........** **..........** **..........** **..........** **..........** **..........** **..........** **..........** **..........** **..........**

**Ohio/113461-3/2005**  **..........** **..........** **..........** **..........** **..........** **.........R** **..........** **..........** **..........** **..........**

**Tottori/1/2007**  **..........** **..........** **..........** **..........** **..........** **..........** **..........** **..........** **..........** **..........**

**Lincolnshire/1/2007**  **..........** **..........** **..........** **..........** **..........** **.........R** **..........** **..........** **..........** **..........**

**California/1/2010**  **..........** **..........** **..........** **..........** **..........** **.........R** **......I...** **..........** **...T.I....** **..........**

**Sweden/VIR165837/2011**  **..........** **..........** **..........** **..........** **..........** **.........R** **......I...** **..........** **...T.I....** **..........**

**Kentucky/1/2011**  **..........** **..........** **..........** **..........** **..........** **.........R** **......I...** **..........** **...T.I....** **..........**

**Kyonggi/SA1/2011**  **..........** **..........** **..........** **..........** **..........** **..........** **..........** **..........** **..........** **..........**

**Dubai/1/2012**  **..........** **..........** **..........** **..........** **..........** **.........R** **......I...** **..........** **...T.I....** **..........**

**Rio Grande do Sul/1/2012** **..........** **..........** **..........** **..........** **..........** **.........R** **......I...** **..........** **...T.I....** **..........**

**Tennessee/28B/2014**  **..........** **..........** **..........** **..........** **..........** **.........R** **T.....I...** **..........** **...T.I....** **........R.**

**Montana/9564-1/2015**  **..........** **..........** **..........** **..........** **..........** **.........R** **T.....I...** **..........** **...T.I....** **........R.**

**Malaysia/1/2015**  **..........** **..........** **..........** **..........** **..........** **.........R** **T.....I...** **..........** **...T.I....** **........R.**

**Georgia/121362-16/2016**  **..........** **..........** **..........** **..........** **..........** **.........R** **T.....I...** **..........** **...T.I....** **........R.**

***710 720 730 740 750***

***....|....| ....|....| ....|....| ....|....| ....|....| ....|....***

**South Africa/4/2003**  **NKRYGPALSI** **NELSKLAKGE** **KANVLIGQGD** **VVLVMKRKRD** **SSILTDSQTA** **TKRIRMAIN**

**Ohio/1/2003**  **..........** **..........** **..........** **I.........** **.---------** **---------**

**Ohio/113461-3/2005**  **..........** **..........** **..........** **I.........** **..........** **.........**

**Tottori/1/2007**  **..........** **..........** **..........** **..........** **..........** **.........**

**Lincolnshire/1/2007**  **..........** **..........** **..........** **..........** **..........** **.........**

**California/1/2010**  **..........** **..........** **..........** **..........** **..........** **.........**

**Sweden/VIR165837/2011**  **..........** **..........** **..........** **..........** **..L.......** **.........**

**Kentucky/1/2011**  **..........** **..........** **..........** **..........** **..........** **.........**

**Kyonggi/SA1/2011**  **..........** **..........** **..........** **..........** **..........** **.........**

**Dubai/1/2012**  **..........** **..........** **..........** **..........** **..........** **.........**

**Rio Grande do Sul/1/2012** **..........** **..........** **..........** **..........** **..........** **.........**

**Tennessee/28B/2014**  **..........** **..........** **..........** **..........** **..........** **.........**

**Montana/9564-1/2015**  **..........** **..........** **..........** **..........** **..........** **...V.....**

**Malaysia/1/2015**  **..........** **..........** **..........** **..........** **..........** **...V.....**

**Georgia/121362-16/2016**  **..........** **..........** **..........** **..........** **..........** **...V.....**

# **Figure S1.2** PB1 Alignment

***10 20 30 40 50 60 70 80 90 100***

***....|....| ....|....| ....|....| ....|....| ....|....| ....|....| ....|....| ....|....| ....|....| ....|....|***

**Ohio/1/2003**  **MDVNPTLLFL** **KVPAQNAIST** **TFPYTGDPPY** **SHGTGTGYTM** **DTVNRTHQYS** **EKGKWTTNTE** **IGAPQLNPID** **GPLPEDNEPS** **GYAQTDCVLE** **AMAFLEESHP**

**Ohio/113461-3/2005**  **..........** **..........** **..........** **..........** **..........** **..........** **..........** **..........** **..........** **...L......**

**Tottori/1/2007**  **..........** **..........** **..........** **..........** **..........** **..........** **..........** **..........** **..........** **...L......**

**Lincolnshire/1/2007**  **--........** **..........** **..........** **..........** **..........** **..........** **..........** **..........** **..........** **...L......**

**California/1/2010**  **..........** **..........** **..........** **..........** **..........** **..........** **..........** **..........** **..........** **...L......**

**Sweden/VIR165837/2011**  **-.........** **..........** **..........** **..........** **..........** **..........** **..........** **..........** **..........** **..VL......**

**Kentucky/1/2011**  **..........** **..........** **..........** **..........** **..........** **..........** **..........** **..........** **..........** **...L......**

**Kyonggi/SA1/2011**  **..........** **..Q.......** **..........** **..........** **..........** **..........** **..........** **..........** **..........** **...L......**

**Dubai/1/2012**  **..........** **..........** **..........** **..........** **..........** **..........** **..........** **..........** **..........** **...L......**

**Rio Grande do Sul/1/2012** **..........** **..........** **..........** **..........** **..........** **..........** **..........** **..........** **..........** **...L......**

**Tennessee/28B/2014**  **..........** **..........** **..........** **..........** **..........** **..........** **..........** **..........** **..........** **...L......**

**Montana/9564-1/2015**  **..........** **..........** **..........** **..........** **..........** **..........** **..........** **..........** **..........** **...L......**

**Malaysia/1/2015**  **..........** **..........** **..........** **..........** **..........** **..........** **..........** **..........** **..........** **...L......**

**Georgia/121362-16/2016**  **..........** **..........** **..........** **..........** **..........** **..........** **..........** **..........** **..........** **...L......**

***110 120 130 140 150 160 170 180 190 200***

***....|....| ....|....| ....|....| ....|....| ....|....| ....|....| ....|....| ....|....| ....|....| ....|....|***

**Ohio/1/2003**  **GIFENSCLET** **MEVIQQTRVD** **KLTQGRQTYD** **WTLNRNQPAA** **TALANTIEVF** **RSNGLTSNES** **GRLMDFLKDV** **MESMNKEEME** **ITTHFQRKRR** **VRDNMTKRMV**

**Ohio/113461-3/2005**  **..........** **..........** **..........** **..........** **..........** **..........** **..........** **..........** **..........** **..........**

**Tottori/1/2007**  **..........** **..........** **..........** **..........** **..........** **..........** **..........** **..........** **..........** **..........**

**Lincolnshire/1/2007**  **..........** **..........** **..........** **..........** **........I.** **..........** **..........** **........I.** **..........** **..........**

**California/1/2010**  **..........** **..........** **..........** **..........** **..........** **..........** **..........** **..........** **..........** **.........I**

**Sweden/VIR165837/2011**  **..........** **..........** **..........** **..........** **...G......** **..........** **..........** **..........** **..........** **.........I**

**Kentucky/1/2011**  **..........** **..........** **..........** **..........** **..........** **..........** **..........** **..........** **..........** **.........I**

**Kyonggi/SA1/2011**  **..........** **..........** **..........** **..........** **..........** **..........** **..........** **..........** **..........** **..........**

**Dubai/1/2012**  **..........** **..........** **..........** **..........** **..........** **..........** **..........** **..........** **..........** **.........I**

**Rio Grande do Sul/1/2012** **..........** **..........** **..........** **..........** **..........** **..........** **..........** **..........** **..........** **.........I**

**Tennessee/28B/2014**  **..........** **...V......** **..........** **..........** **..........** **..........** **..........** **..........** **..........** **.........I**

**Montana/9564-1/2015**  **..........** **...V......** **..........** **..........** **..........** **..........** **..........** **..........** **..........** **.........I**

**Malaysia/1/2015**  **..........** **...V......** **..........** **..........** **..........** **..........** **..........** **..........** **..........** **.........I**

**Georgia/121362-16/2016**  **..........** **...V......** **..........** **..........** **..........** **..........** **..........** **..........** **..........** **.........I**

***210 220 230 240 250 260 270 280 290 300***

***....|....| ....|....| ....|....| ....|....| ....|....| ....|....| ....|....| ....|....| ....|....| ....|....|***

**Ohio/1/2003**  **TQRTIGKKKQ** **RLNRKSYLIR** **TLTLNTMTKD** **AERGKLKRRA** **IATPGMQIRG** **FVYFVETLAR** **RICEKLEQSG** **LPVGGNEKKA** **KLANVVRKMM** **TNSQDTELSF**

**Ohio/113461-3/2005**  **..........** **..........** **..........** **..........** **..........** **..........** **..........** **..........** **..........** **..........**

**Tottori/1/2007**  **..........** **..........** **..........** **..........** **..........** **..........** **..........** **..........** **..........** **..........**

**Lincolnshire/1/2007**  **..........** **..........** **..........** **..........** **..........** **..........** **..........** **..........** **..........** **..........**

**California/1/2010**  **..........** **..........** **..........** **..........** **..........** **..........** **..........** **..........** **..........** **..........**

**Sweden/VIR165837/2011**  **..........** **..........** **..........** **..........** **..........** **..........** **..........** **..........** **..........** **..........**

**Kentucky/1/2011**  **..........** **..........** **..........** **..........** **..........** **..........** **..........** **..........** **..........** **..........**

**Kyonggi/SA1/2011**  **..........** **..........** **..........** **..........** **..........** **..........** **..........** **..........** **..........** **..........**

**Dubai/1/2012**  **..........** **..........** **..........** **..........** **..........** **..........** **..........** **..........** **..........** **..........**

**Rio Grande do Sul/1/2012** **..........** **..........** **..........** **..........** **..........** **..........** **..........** **..........** **..........** **..........**

**Tennessee/28B/2014**  **..........** **..........** **..........** **..........** **..........** **..........** **..........** **..........** **..........** **..........**

**Montana/9564-1/2015**  **..........** **..........** **..........** **..........** **..........** **..........** **..........** **..........** **..........** **..........**

**Malaysia/1/2015**  **..........** **..........** **..........** **..........** **..........** **..........** **..........** **..........** **..........** **..........**

**Georgia/121362-16/2016**  **..........** **..........** **..........** **..........** **..........** **..........** **..........** **..........** **..........** **..........**

***310 320 330 340 350 360 370 380 390 400***

***....|....| ....|....| ....|....| ....|....| ....|....| ....|....| ....|....| ....|....| ....|....| ....|....|***

**Ohio/1/2003**  **TITGDNTKWN** **ENQNPRIFLA** **MITYITRNQP** **EWFRNVLSIA** **PIMFSNKMAR** **LGKGYMFESK** **SMKLRTQIPA** **EMLASIDLKY** **FNDSTKKKIE** **KIRPLLVDGT**

**Ohio/113461-3/2005**  **..........** **..........** **..........** **..........** **..........** **..........** **..........** **..........** **..........** **..........**

**Tottori/1/2007**  **..........** **..........** **..........** **..........** **..........** **..........** **..........** **..........** **..........** **..........**

**Lincolnshire/1/2007**  **..........** **..........** **..........** **..........** **..........** **..........** **..........** **..........** **..........** **..........**

**California/1/2010**  **..........** **..........** **..........** **..........** **..........** **..........** **..........** **..........** **..........** **..........**

**Sweden/VIR165837/2011**  **..........** **..........** **..........** **..........** **..........** **..........** **..........** **..........** **..........** **..........**

**Kentucky/1/2011**  **..........** **..........** **..........** **..........** **..........** **..........** **..........** **..........** **..........** **..........**

**Kyonggi/SA1/2011**  **..........** **..........** **..........** **..........** **..........** **..........** **..........** **..........** **..........** **..........**

**Dubai/1/2012**  **..........** **..........** **..........** **..........** **..........** **..........** **..........** **..........** **..........** **..........**

**Rio Grande do Sul/1/2012** **..........** **..........** **..........** **..........** **..........** **..........** **..........** **..........** **..........** **..........**

**Tennessee/28B/2014**  **..........** **..........** **..........** **..........** **..........** **..........** **..........** **..........** **..........** **..........**

**Montana/9564-1/2015**  **..........** **..........** **..........** **..........** **..........** **..........** **..........** **..........** **..........** **..........**

**Malaysia/1/2015**  **..........** **..........** **..........** **..........** **..........** **..........** **..........** **..........** **..........** **..........**

**Georgia/121362-16/2016**  **..........** **..........** **..........** **..........** **..........** **..........** **..........** **..........** **..........** **..........**

***410 420 430 440 450 460 470 480 490 500***

***....|....| ....|....| ....|....| ....|....| ....|....| ....|....| ....|....| ....|....| ....|....| ....|....|***

**Ohio/1/2003**  **ASLSPGMMMG** **MFNMLSTVLG** **VSILNLGQRK** **YTKTTYWWDG** **LQSSDDFALI** **VNAPNHEGIQ** **AGVDRFYRTC** **KLVGINMSKK** **KSYINRTGTF** **EFTSFFYRYG**

**Ohio/113461-3/2005**  **..........** **..........** **..........** **..........** **..........** **..........** **..........** **..........** **..........** **..........**

**Tottori/1/2007**  **..........** **..........** **..........** **..........** **..........** **..........** **..........** **..........** **..........** **..........**

**Lincolnshire/1/2007**  **..........** **..........** **..........** **..........** **..........** **..........** **..........** **..........** **..........** **..........**

**California/1/2010**  **..........** **..........** **..........** **..........** **..........** **..........** **..........** **..........** **..........** **..........**

**Sweden/VIR165837/2011**  **..........** **..........** **..........** **..........** **..........** **..........** **..........** **.......RQ.** **..........** **..........**

**Kentucky/1/2011**  **..........** **..........** **..........** **..........** **..........** **..........** **..........** **..........** **..........** **..........**

**Kyonggi/SA1/2011**  **..........** **..........** **..........** **..........** **..........** **..........** **..........** **..........** **..........** **..........**

**Dubai/1/2012**  **..........** **..........** **..........** **..........** **..........** **..........** **..........** **..........** **..........** **..........**

**Rio Grande do Sul/1/2012** **..........** **..........** **..........** **..........** **..........** **..........** **..........** **..........** **..........** **..........**

**Tennessee/28B/2014**  **..........** **..........** **..........** **..........** **..........** **..........** **..........** **..........** **..........** **..........**

**Montana/9564-1/2015**  **..........** **..........** **..........** **..........** **..........** **..........** **..........** **..........** **..........** **..........**

**Malaysia/1/2015**  **..........** **..........** **..........** **..........** **..........** **..........** **..........** **..........** **..........** **..........**

**Georgia/121362-16/2016**  **..........** **..........** **..........** **..........** **..........** **..........** **..........** **..........** **..........** **..........**

***510 520 530 540 550 560 570 580 590 600***

***....|....| ....|....| ....|....| ....|....| ....|....| ....|....| ....|....| ....|....| ....|....| ....|....|***

**Ohio/1/2003**  **FVANFSMELP** **SFGVSGINES** **ADMSIGVTVI** **KNNMINNDLG** **PATAQMALQL** **FIKDYRYTYR** **CHRGDTQIQT** **RRSFELKKLW** **EQTRSKTGLL** **VSDGGPNLYN**

**Ohio/113461-3/2005**  **..........** **..........** **..........** **..........** **..........** **..........** **..........** **.......I..** **..........** **..........**

**Tottori/1/2007**  **..........** **..........** **..........** **..........** **..........** **..........** **..........** **..........** **..........** **..........**

**Lincolnshire/1/2007**  **..........** **..........** **..........** **..........** **..........** **..........** **..........** **..........** **..........** **..........**

**California/1/2010**  **..........** **..........** **..........** **..........** **..........** **..........** **..........** **..........** **...Q......** **..........**

**Sweden/VIR165837/2011**  **..........** **..........** **..........** **..........** **..........** **..........** **..........** **..........** **...Q......** **..........**

**Kentucky/1/2011**  **..........** **..........** **..........** **..........** **..........** **..........** **..........** **..........** **...Q......** **..........**

**Kyonggi/SA1/2011**  **..........** **..........** **..........** **..........** **..........** **..........** **..........** **..........** **..........** **..........**

**Dubai/1/2012**  **..........** **..........** **..........** **..........** **..........** **..........** **..........** **..........** **...Q......** **..........**

**Rio Grande do Sul/1/2012** **..........** **..........** **..........** **..........** **..........** **..........** **..........** **..........** **...Q......** **..........**

**Tennessee/28B/2014**  **..........** **..........** **..........** **..........** **..........** **..........** **..........** **..........** **...Q......** **..........**

**Montana/9564-1/2015**  **..........** **..........** **..........** **..........** **..........** **..........** **..........** **..........** **...Q......** **..........**

**Malaysia/1/2015**  **..........** **..........** **..........** **..........** **..........** **..........** **..........** **..........** **...Q......** **..........**

**Georgia/121362-16/2016**  **..........** **..........** **..........** **..........** **..........** **..........** **..........** **..........** **...Q......** **..........**

***610 620 630 640 650 660 670 680 690 700***

***....|....| ....|....| ....|....| ....|....| ....|....| ....|....| ....|....| ....|....| ....|....| ....|....|***

**Ohio/1/2003**  **IRNLHIPEVC** **LKWELMDEDY** **KGRLCNPLNP** **FVSHKEIESV** **NSAVVMPAHG** **PAKSMEYDAV** **ATTHSWIPKR** **NRSILNTSQR** **GILEDEQMYQ** **KCCNLFEKFF**

**Ohio/113461-3/2005**  **..........** **..........** **R.........** **..........** **..........** **..........** **..........** **..........** **..........** **..........**

**Tottori/1/2007**  **..........** **..........** **R.........** **..........** **..........** **...N......** **..........** **..........** **..........** **..........**

**Lincolnshire/1/2007**  **..........** **..........** **R.........** **..........** **..........** **..........** **..........** **..........** **..........** **..........**

**California/1/2010**  **..........** **..........** **R.........** **..........** **..........** **..........** **..........** **..........** **..........** **..........**

**Sweden/VIR165837/2011**  **..........** **..........** **R.........** **..........** **...I......** **..........** **..........** **..........** **..........** **..........**

**Kentucky/1/2011**  **..........** **..........** **R.........** **..........** **...I......** **..........** **..........** **..........** **..........** **..........**

**Kyonggi/SA1/2011**  **..........** **..........** **R.........** **..........** **..........** **..........** **..........** **..........** **..........** **..........**

**Dubai/1/2012**  **..........** **..........** **R.........** **..........** **...I......** **..........** **..........** **..........** **..........** **..........**

**Rio Grande do Sul/1/2012** **..........** **..........** **R.........** **..........** **...I......** **..........** **..........** **..........** **..........** **..........**

**Tennessee/28B/2014**  **..........** **..........** **R.........** **..........** **...I......** **..........** **..........** **..........** **..........** **..........**

**Montana/9564-1/2015**  **..........** **..........** **R.........** **..........** **...I......** **..........** **..........** **..........** **..........** **..........**

**Malaysia/1/2015**  **..........** **..........** **R.........** **..........** **...I......** **..........** **..........** **..........** **..........** **..........**

**Georgia/121362-16/2016**  **..........** **..........** **R.........** **..........** **...I......** **..........** **..........** **..........** **..........** **..........**

***710 720 730 740 750***

***....|....| ....|....| ....|....| ....|....| ....|....| ....|..***

**Ohio/1/2003**  **PSSSYRRPVG** **ISSMVEAMVS** **RARIDARIDF** **ESGRIKKDEF** **AEIMKICSTI** **EELRRQK**

**Ohio/113461-3/2005**  **..........** **..........** **..........** **..........** **..........** **.......**

**Tottori/1/2007**  **..........** **..........** **..........** **..........** **..........** **.......**

**Lincolnshire/1/2007**  **..........** **..........** **..........** **..........** **..........** **.......**

**California/1/2010**  **..........** **..........** **..........** **..........** **..........** **.......**

**Sweden/VIR165837/2011**  **..........** **..........** **..........** **..........** **..........** **.......**

**Kentucky/1/2011**  **..........** **..........** **..........** **..........** **..........** **.......**

**Kyonggi/SA1/2011**  **..........** **..........** **..........** **..........** **..........** **.......**

**Dubai/1/2012**  **..........** **..........** **..........** **..........** **..........** **.......**

**Rio Grande do Sul/1/2012** **..........** **..........** **..........** **..........** **..........** **.......**

**Tennessee/28B/2014**  **..........** **....A.....** **..........** **..........** **..........** **...G...**

**Montana/9564-1/2015**  **..........** **....A.....** **..........** **..........** **..........** **...G...**

**Malaysia/1/2015**  **..........** **....A.....** **..........** **..........** **..........** **...G...**

**Georgia/121362-16/2016**  **..........** **....A.....** **..........** **..........** **..........** **...G...**

# **Figure S1.3** PB1-F2 Alignment

***10 20 30 40 50 60 70 80 90***

***....|....| ....|....| ....|....| ....|....| ....|....| ....|....| ....|....| ....|....| ....|....|***

**Ohio/1/2003**  **MEQGQDTPWI** **LSTEHTNIQK** **KGNGQQTLRL** **EHHNLIQSMD** **HFLKTMNQVG** **TPKQIVYWKQ** **WLSLKNPIPE** **SLKIRVLKRW** **R**

**Ohio/113461-3/2005**  **..........** **..........** **..........** **..........** **..........** **..........** **..Y.......** **..........** **.**

**Tottori/1/2007**  **..........** **..........** **..........** **..........** **..........** **..........** **..Y......G** **..........** **.**

**Lincolnshire/1/2007**  **..........** **..........** **..........** **..........** **..........** **..........** **..Y.......** **..........** **.**

**California/1/2010**  **..........** **..........** **..........** **..........** **..........** **..........** **..Y.......** **..........** **.**

**Sweden/VIR165837/2011**  **..........** **..........** **R.........** **..........** **.........D** **..........** **.FY.......** **..........** **.**

**Kentucky/1/2011**  **..........** **..........** **..........** **..........** **.........D** **..........** **..Y.......** **..........** **.**

**Kyonggi/SA1/2011**  **..........** **..........** **..........** **..........** **..........** **..........** **..Y......G** **..........** **.**

**Dubai/1/2012**  **..........** **..........** **..........** **..........** **.........D** **..........** **..Y.......** **..........** **.**

**Rio Grande do Sul/1/2012** **..........** **..........** **..........** **..........** **.........D** **..........** **..Y.......** **..........** **.**

**Tennessee/28B/2014**  **..........** **..........** **..........** **..........** **.........D** **..........** **..Y.......** **........Q.** **.WYSRQEWTN**

**Montana/9564-1/2015**  **..........** **..........** **..........** **..........** **.........D** **..........** **..Y.......** **........Q.** **.WYSRQEWTN**

**Malaysia/1/2015**  **..........** **..........** **..........** **..........** **.........D** **..........** **..Y.......** **........Q.** **.WYSRQEWTN**

**Georgia/121362-16/2016**  **..........** **..........** **..........** **..........** **.........D** **..........** **..Y.......** **........Q.** **.WYNRQEWTN**

# **Figure S1.4** PA Alignment

***10 20 30 40 50 60 70 80 90 100***

***....|....| ....|....| ....|....| ....|....| ....|....| ....|....| ....|....| ....|....| ....|....| ....|....|***

**South Africa/4/2003**  **MEDFVRQCFN** **PMIVELAEKA** **MKEYGEDPKI** **ETNKFAAICT** **HLEVCFMYSD** **FHFINELGES** **VVIESGDPNA** **LLKHRFEIIE** **GRDRTMAWTV** **VNSICNTTRA**

**Ohio/1/2003**  **..........** **..........** **..........** **..........** **..........** **..........** **..........** **..........** **..........** **..........**

**Ohio/113461-3/2005**  **..........** **..........** **..........** **..........** **..........** **..........** **..........** **..........** **..........** **..........**

**Tottori/1/2007**  **..........** **..........** **..........** **..........** **..........** **..........** **..........** **..........** **..........** **..........**

**Lincolnshire/1/2007**  **..........** **..........** **..........** **..........** **..........** **..........** **..........** **..........** **..........** **..........**

**California/1/2010**  **..........** **..........** **..........** **..........** **..........** **..........** **..........** **..........** **..........** **..........**

**Sweden/VIR165837/2011**  **-.........** **........N.** **..........** **..........** **..........** **........K.** **..........** **..........** **..........** **..........**

**Kentucky/1/2011**  **..........** **..........** **..........** **..........** **..........** **........K.** **..........** **..........** **..........** **..........**

**Kyonggi/SA1/2011**  **..........** **..........** **..........** **..........** **..........** **..........** **..........** **..........** **..........** **..........**

**Dubai/1/2012_PA**  **..........** **..........** **..........** **..........** **..........** **........K.** **..........** **..........** **..........** **..........**

**Rio Grande do Sul/1/2012** **..........** **..........** **..........** **..........** **..........** **........K.** **..........** **..........** **..........** **..........**

**Tennessee/28B/2014**  **..........** **..........** **..........** **..........** **..........** **........K.** **..........** **..........** **..........** **..........**

**Montana/9564-1/2015**  **..........** **..........** **..........** **..........** **..........** **........K.** **..........** **..........** **..........** **..........**

**Malaysia/1/2015**  **..........** **..........** **..........** **..........** **..........** **........K.** **..........** **..........** **..........** **..........**

**Georgia/121362-16/2016**  **..........** **..........** **..........** **..........** **..........** **........K.** **..........** **..........** **..........** **..........**

***110 120 130 140 150 160 170 180 190 200***

***....|....| ....|....| ....|....| ....|....| ....|....| ....|....| ....|....| ....|....| ....|....| ....|....|***

**South Africa/4/2003**  **EKPKFLPDLY** **DYKENRFVEI** **GVTRREVHIY** **YLEKANKIKS** **EKTHIHIFSF** **TGEEMATKAD** **YTLDEESRAR** **IKTRLFTIRQ** **EMASRGLWDS** **FRQSERGEET**

**Ohio/1/2003**  **..........** **..........** **..........** **..........** **..........** **..........** **..........** **..........** **..........** **..........**

**Ohio/113461-3/2005**  **..........** **..........** **..........** **..........** **..........** **..........** **..........** **..........** **..........** **..........**

**Tottori/1/2007**  **..........** **..........** **..........** **..........** **..........** **..........** **..........** **..........** **..........** **..........**

**Lincolnshire/1/2007**  **..........** **..........** **..........** **..........** **..........** **..........** **..........** **..........** **..........** **..........**

**California/1/2010**  **..........** **..........** **..........** **..........** **..........** **..........** **..........** **..........** **..........** **..........**

**Sweden/VIR165837/2011**  **..........** **..........** **..........** **..........** **..........** **..........** **..........** **..........** **..........** **..........**

**Kentucky/1/2011**  **..........** **..........** **..........** **..........** **..........** **..........** **..........** **..........** **..........** **..........**

**Kyonggi/SA1/2011**  **..........** **..........** **..........** **..........** **..........** **..........** **..........** **..........** **..........** **..........**

**Dubai/1/2012_PA**  **..........** **..........** **..........** **..........** **..........** **..........** **..........** **..........** **..........** **..........**

**Rio Grande do Sul/1/2012** **..........** **..........** **..........** **..........** **..........** **..........** **..........** **..........** **..........** **..........**

**Tennessee/28B/2014**  **..........** **..........** **..........** **..........** **..........** **..........** **..........** **..........** **..........** **..........**

**Montana/9564-1/2015**  **..........** **..........** **..........** **..........** **..........** **..........** **..........** **..........** **..........** **..........**

**Malaysia/1/2015**  **..........** **..........** **..........** **..........** **..........** **..........** **..........** **..........** **..........** **..........**

**Georgia/121362-16/2016**  **..........** **..........** **..........** **..........** **..........** **..........** **..........** **..........** **..........** **..........**

***210 220 230 240 250 260 270 280 290 300***

***....|....| ....|....| ....|....| ....|....| ....|....| ....|....| ....|....| ....|....| ....|....| ....|....|***

**South Africa/4/2003**  **IEERFEITGT** **MRKLANYSLP** **PNFSSLENFR** **VYVDGFEPNG** **CIESKLSQMS** **KEVNARIEPF** **SKTTPRPLKM** **PGGPPCHQRS** **KFLLMDALKL** **SIEDPSHEGE**

**Ohio/1/2003**  **..........** **..........** **..........** **..........** **..........** **..........** **..........** **..........** **..........** **..........**

**Ohio/113461-3/2005**  **..........** **..........** **..........** **..........** **..........** **..........** **..........** **..........** **..........** **..........**

**Tottori/1/2007**  **..........** **..........** **..........** **..........** **..........** **........S.** **..........** **..........** **..........** **..........**

**Lincolnshire/1/2007**  **.........A** **..........** **..........** **.........E** **..........** **........S.** **..........** **..........** **..........** **..........**

**California/1/2010**  **..........** **..........** **..........** **..........** **..........** **........S.** **..........** **..........** **..........** **..........**

**Sweden/VIR165837/2011**  **..........** **..........** **..........** **......K...** **..........** **........S.** **..........** **..........** **..........** **..........**

**Kentucky/1/2011**  **..........** **..........** **..........** **..........** **..........** **........S.** **..........** **..........** **..........** **..........**

**Kyonggi/SA1/2011**  **..........** **..........** **..........** **..........** **..........** **........S.** **..........** **..........** **..........** **..........**

**Dubai/1/2012_PA**  **..........** **..........** **..........** **..........** **..........** **........S.** **..........** **..........** **..........** **..........**

**Rio Grande do Sul/1/2012** **..........** **..........** **..........** **..........** **..........** **........S.** **..........** **..........** **..........** **..........**

**Tennessee/28B/2014**  **..........** **..........** **..........** **......K...** **..........** **........S.** **..........** **..........** **..........** **..........**

**Montana/9564-1/2015**  **..........** **..........** **..........** **......K...** **..........** **........S.** **..........** **..........** **..........** **..........**

**Malaysia/1/2015**  **..........** **..........** **..........** **......K...** **..........** **........S.** **..........** **..........** **..........** **..........**

**Georgia/121362-16/2016**  **..........** **..........** **..........** **......K...** **..........** **........S.** **..........** **..........** **..........** **..........**

***310 320 330 340 350 360 370 380 390 400***

***....|....| ....|....| ....|....| ....|....| ....|....| ....|....| ....|....| ....|....| ....|....| ....|....|***

**South Africa/4/2003**  **GIPLYDAIKC** **MKTFFGWKEP** **SIVKPHEKGI** **NPNYLQTWKQ** **VLEEIQDLEN** **EERTPKTKNM** **KKTSQLKWAL** **GENMAPEKVD** **FEDCKDISDL** **KQYDSDEPET**

**Ohio/1/2003**  **..........** **..........** **..........** **..........** **..........** **..........** **..........** **..........** **..........** **..........**

**Ohio/113461-3/2005**  **..........** **..........** **..........** **..........** **..........** **..........** **..........** **..........** **..........** **..........**

**Tottori/1/2007**  **..........** **..........** **..........** **..........** **..........** **..........** **..........** **..........** **..........** **..........**

**Lincolnshire/1/2007**  **..........** **..........** **..........** **..........** **.......I..** **...I......** **..........** **..........** **..........** **..........**

**California/1/2010**  **..........** **..........** **..........** **..........** **.......I..** **...I......** **..........** **..........** **..........** **..........**

**Sweden/VIR165837/2011**  **..........** **..........** **..........** **..........** **.......I..** **...I......** **..........** **..........** **..........** **..........**

**Kentucky/1/2011**  **..........** **..........** **..........** **..........** **.......I..** **...I......** **..........** **..........** **..........** **..........**

**Kyonggi/SA1/2011**  **..........** **..........** **..........** **..........** **..........** **..........** **..........** **..........** **..........** **..........**

**Dubai/1/2012_PA**  **..........** **..........** **..........** **..........** **.......I..** **...I......** **..........** **..........** **..........** **..........**

**Rio Grande do Sul/1/2012** **..........** **..........** **..........** **..........** **.......I..** **...I......** **..........** **..........** **..........** **..........**

**Tennessee/28B/2014**  **..........** **..........** **..........** **..........** **.......I..** **...I......** **..........** **..........** **..........** **..........**

**Montana/9564-1/2015**  **..........** **..........** **..........** **..........** **.......I..** **...I......** **..........** **..........** **..........** **..........**

**Malaysia/1/2015**  **..........** **..........** **..........** **..........** **.......I..** **...I......** **..........** **..........** **....R.....** **..........**

**Georgia/121362-16/2016**  **..........** **..........** **..........** **....I.....** **.......I..** **...I......** **..........** **..........** **..........** **..........**

***410 420 430 440 450 460 470 480 490 500***

***....|....| ....|....| ....|....| ....|....| ....|....| ....|....| ....|....| ....|....| ....|....| ....|....|***

**South Africa/4/2003**  **RSLASWIQSE** **FNKACELTDS** **SWIELDEIGE** **DVAPIEYIAS** **MRRNYFTAEI** **SHCRATEYIM** **KGVYINTALL** **NASCAAMDEF** **QLIPMISKCR** **TKEGRRKTNL**

**Ohio/1/2003**  **..........** **..........** **..........** **..........** **..........** **..........** **..........** **..........** **..........** **..........**

**Ohio/113461-3/2005**  **..........** **..........** **..........** **..........** **..........** **..........** **....V.....** **..........** **..........** **..........**

**Tottori/1/2007**  **........N.** **..........** **..........** **.I........** **..........** **..........** **....V.....** **..........** **..........** **..........**

**Lincolnshire/1/2007**  **........N.** **..........** **..........** **..........** **..........** **..........** **....V.....** **..........** **..........** **..........**

**California/1/2010**  **........N.** **..........** **..........** **..........** **..........** **..........** **....V.....** **..........** **..........** **..........**

**Sweden/VIR165837/2011**  **........N.** **..........** **..........** **..........** **..........** **..........** **....V.....** **..........** **..........** **..........**

**Kentucky/1/2011**  **........N.** **..........** **..........** **..........** **..........** **..........** **....V.....** **..........** **..........** **..........**

**Kyonggi/SA1/2011**  **........N.** **..........** **..........** **..........** **..........** **..........** **....V.....** **..........** **..........** **..........**

**Dubai/1/2012_PA**  **........N.** **..........** **..........** **..........** **..........** **..........** **....V.....** **..........** **..........** **..........**

**Rio Grande do Sul/1/2012** **........N.** **..........** **..........** **..........** **..........** **..........** **....V.....** **..........** **..........** **..........**

**Tennessee/28B/2014**  **........N.** **..........** **..........** **..........** **..........** **..........** **....V.....** **..........** **..........** **..........**

**Montana/9564-1/2015**  **........N.** **..........** **..........** **..........** **..........** **..........** **....V.....** **..........** **..........** **..........**

**Malaysia/1/2015**  **........N.** **..........** **..........** **..........** **..........** **..........** **....V.....** **..........** **..........** **..........**

**Georgia/121362-16/2016**  **........N.** **..........** **..........** **..........** **..........** **..........** **....V.....** **..........** **..........** **..........**

***510 520 530 540 550 560 570 580 590 600***

***....|....| ....|....| ....|....| ....|....| ....|....| ....|....| ....|....| ....|....| ....|....| ....|....|***

**South Africa/4/2003**  **YGFIIKGRSH** **LRNDTDVVNF** **VSMEFSLTDP** **RFEPHKWEKY** **CVLEIGDMLL** **RTAVGQVSRP** **MFLYVRTNGT** **SKIKMKWGME** **MRRCLLQSLQ** **QIESMIEAES**

**Ohio/1/2003**  **....V.....** **..........** **..........** **..........** **..........** **..........** **..........** **..........** **..........** **..........**

**Ohio/113461-3/2005**  **....V.....** **..........** **..........** **..........** **..........** **..........** **..........** **..........** **..........** **..........**

**Tottori/1/2007**  **....V.....** **..........** **..........** **..........** **..........** **..........** **..........** **..........** **..........** **..........**

**Lincolnshire/1/2007**  **....V.....** **..........** **..........** **..........** **..........** **..........** **..........** **..........** **..........** **..........**

**California/1/2010**  **....V.....** **..........** **..........** **..........** **..........** **..........** **..........** **..........** **..........** **..........**

**Sweden/VIR165837/2011**  **....V.....** **..........** **..........** **..........** **..........** **..........** **..........** **..........** **..........** **..........**

**Kentucky/1/2011**  **....V.....** **..........** **..........** **..........** **..........** **..........** **..........** **..........** **..........** **..........**

**Kyonggi/SA1/2011**  **....V.....** **..........** **..........** **..........** **..........** **..........** **..........** **..........** **..........** **..........**

**Dubai/1/2012_PA**  **....V.....** **..........** **..........** **..........** **..........** **..........** **..........** **..........** **..........** **..........**

**Rio Grande do Sul/1/2012** **....V.....** **..........** **..........** **..........** **..........** **..........** **..........** **..........** **..........** **..........**

**Tennessee/28B/2014**  **....V.....** **..........** **..........** **..........** **..........** **..........** **..........** **..........** **..........** **..........**

**Montana/9564-1/2015**  **....V.....** **..........** **..........** **..........** **..........** **..........** **..........** **..........** **..........** **..........**

**Malaysia/1/2015**  **....V.....** **..........** **..........** **..........** **..........** **..........** **..........** **..........** **..........** **..........**

**Georgia/121362-16/2016**  **....V.....** **..........** **..........** **..........** **..........** **..........** **..........** **..........** **..........** **..........**

***610 620 630 640 650 660 670 680 690 700***

***....|....| ....|....| ....|....| ....|....| ....|....| ....|....| ....|....| ....|....| ....|....| ....|....|***

**South Africa/4/2003**  **SVKEKDMTKE** **FFENKSETWP** **IGESPKGVEE** **GSIGKVCRTL** **LAKSVFNSLY** **ASPQLEGFSA** **ESRKLLLIVQ** **ALRDNLEPGT** **FDIGGLYESI** **EECLINDPWV**

**Ohio/1/2003**  **..........** **..........** **..........** **..........** **..........** **..........** **..........** **..........** **..........** **..........**

**Ohio/113461-3/2005**  **..........** **..........** **..........** **..........** **..........** **..........** **..........** **..........** **..........** **..........**

**Tottori/1/2007**  **..........** **..........** **..........** **..........** **..........** **..........** **..........** **..........** **...E......** **..........**

**Lincolnshire/1/2007**  **..........** **..........** **..........** **..........** **..........** **..........** **..........** **..........** **..........** **..........**

**California/1/2010**  **..........** **..........** **..........** **..........** **..........** **..........** **..........** **..........** **..........** **..........**

**Sweden/VIR165837/2011**  **..........** **..........** **..........** **..........** **..........** **..........** **..........** **..........** **..........** **..........**

**Kentucky/1/2011**  **..........** **..........** **..........** **..........** **..........** **..........** **..........** **..........** **..........** **..........**

**Kyonggi/SA1/2011**  **..........** **..........** **..........** **..........** **..........** **..........** **..........** **..........** **...E......** **..........**

**Dubai/1/2012_PA**  **..........** **..........** **..........** **..........** **..........** **..........** **..........** **..........** **..........** **..........**

**Rio Grande do Sul/1/2012** **..........** **..........** **..........** **..........** **..........** **..........** **..........** **..........** **..........** **..........**

**Tennessee/28B/2014**  **..........** **..........** **.......L..** **..........** **..........** **..........** **..........** **..........** **..........** **..........**

**Montana/9564-1/2015**  **..........** **..........** **..........** **..........** **..........** **..........** **..........** **..........** **..........** **..........**

**Malaysia/1/2015**  **..........** **..........** **..........** **..........** **..........** **..........** **..........** **..........** **..........** **..........**

**Georgia/121362-16/2016**  **..........** **..........** **..........** **..........** **..........** **..........** **..........** **..........** **..........** **..........**

***710***

***....|....| ....|.***

**South Africa/4/2003**  **LLNASWFNSF** **LTHALK**

**Ohio/1/2003**  **..--------** **------**

**Ohio/113461-3/2005**  **..........** **......**

**Tottori/1/2007**  **..........** **......**

**Lincolnshire/1/2007**  **..........** **......**

**California/1/2010**  **..........** **......**

**Sweden/VIR165837/2011**  **..........** **......**

**Kentucky/1/2011**  **..........** **......**

**Kyonggi/SA1/2011**  **..........** **......**

**Dubai/1/2012_PA**  **..........** **......**

**Rio Grande do Sul/1/2012** **..........** **......**

**Tennessee/28B/2014**  **..........** **......**

**Montana/9564-1/2015**  **..........** **......**

**Malaysia/1/2015**  **..........** **......**

**Georgia/121362-16/2016**  **..........** **......**

# **Figure S1.5 PA-X Alignment**

***10 20 30 40 50 60 70 80 90 100***

***....|....| ....|....| ....|....| ....|....| ....|....| ....|....| ....|....| ....|....| ....|....| ....|....|***

**South Africa/4/2003**  **MEDFVRQCFN** **PMIVELAEKA** **MKEYGEDPKI** **ETNKFAAICT** **HLEVCFMYSD** **FHFINELGES** **VVIESGDPNA** **LLKHRFEIIE** **GRDRTMAWTV** **VNSICNTTRA**

**Ohio/1/2003**  **..........** **..........** **..........** **..........** **..........** **..........** **..........** **..........** **..........** **..........**

**Ohio/113461-3/2005**  **..........** **..........** **..........** **..........** **..........** **..........** **..........** **..........** **..........** **..........**

**Tottori/1/2007**  **..........** **..........** **..........** **..........** **..........** **..........** **..........** **..........** **..........** **..........**

**Lincolnshire/1/2007**  **..........** **..........** **..........** **..........** **..........** **..........** **..........** **..........** **..........** **..........**

**California/1/2010**  **..........** **..........** **..........** **..........** **..........** **..........** **..........** **..........** **..........** **..........**

**Sweden/VIR165837/2011**  **..........** **........N.** **..........** **..........** **..........** **........K.** **..........** **..........** **..........** **..........**

**Kentucky/1/2011**  **..........** **..........** **..........** **..........** **..........** **........K.** **..........** **..........** **..........** **..........**

**Kyonggi/SA1/2011**  **..........** **..........** **..........** **..........** **..........** **..........** **..........** **..........** **..........** **..........**

**Dubai/1/2012**  **..........** **..........** **..........** **..........** **..........** **........K.** **..........** **..........** **..........** **..........**

**Rio Grande do Sul/1/2012** **..........** **..........** **..........** **..........** **..........** **........K.** **..........** **..........** **..........** **..........**

**Tennessee/28B/2014**  **..........** **..........** **..........** **..........** **..........** **........K.** **..........** **..........** **..........** **..........**

**Montana/9564-1/2015**  **..........** **..........** **..........** **..........** **..........** **........K.** **..........** **..........** **..........** **..........**

**Malaysia/1/2015**  **..........** **..........** **..........** **..........** **..........** **........K.** **..........** **..........** **..........** **..........**

**Georgia/121362-16/2016**  **..........** **..........** **..........** **..........** **..........** **........K.** **..........** **..........** **..........** **..........**

***110 120 130 140 150 160 170 180 190 200***

***....|....| ....|....| ....|....| ....|....| ....|....| ....|....| ....|....| ....|....| ....|....| ....|....|***

**South Africa/4/2003**  **EKPKFLPDLY** **DYKENRFVEI** **GVTRREVHIY** **YLEKANKIKS** **EKTHIHIFSF** **TGEEMATKAD** **YTLDEESRAR** **IKTRLFTIRQ** **EMASRGLWDS** **FVSPREAKRQ**

**Ohio/1/2003**  **..........** **..........** **..........** **..........** **..........** **..........** **..........** **..........** **..........** **..........**

**Ohio/113461-3/2005**  **..........** **..........** **..........** **..........** **..........** **..........** **..........** **..........** **..........** **..........**

**Tottori/1/2007**  **..........** **..........** **..........** **..........** **..........** **..........** **..........** **..........** **..........** **..........**

**Lincolnshire/1/2007**  **..........** **..........** **..........** **..........** **..........** **..........** **..........** **..........** **..........** **..........**

**California/1/2010**  **..........** **..........** **..........** **..........** **..........** **..........** **..........** **..........** **..........** **..........**

**Sweden/VIR165837/2011**  **..........** **..........** **..........** **..........** **..........** **..........** **..........** **..........** **..........** **..........**

**Kentucky/1/2011**  **..........** **..........** **..........** **..........** **..........** **..........** **..........** **..........** **..........** **..........**

**Kyonggi/SA1/2011**  **..........** **..........** **..........** **..........** **..........** **..........** **..........** **..........** **..........** **..........**

**Dubai/1/2012**  **..........** **..........** **..........** **..........** **..........** **..........** **..........** **..........** **..........** **..........**

**Rio Grande do Sul/1/2012** **..........** **..........** **..........** **..........** **..........** **..........** **..........** **..........** **..........** **..........**

**Tennessee/28B/2014**  **..........** **..........** **..........** **..........** **..........** **..........** **..........** **..........** **..........** **..........**

**Montana/9564-1/2015**  **..........** **..........** **..........** **..........** **..........** **..........** **..........** **..........** **..........** **..........**

**Malaysia/1/2015**  **..........** **..........** **..........** **..........** **..........** **..........** **..........** **..........** **..........** **..........**

**Georgia/121362-16/2016**  **..........** **..........** **..........** **..........** **..........** **..........** **..........** **..........** **..........** **..........**

***210 220 230 240 250***

***....|....| ....|....| ....|....| ....|....| ....|....| ..***

**South Africa/4/2003**  **LKKDLKSQGR** **CASLPITVSH** **RTSPALKILE** **SMWMDSNRTA** **ALRVSFLKCP** **KK**

**Ohio/1/2003**  **..........** **..........** **..........** **..........** **..........** **..**

**Ohio/113461-3/2005**  **..........** **..........** **..........** **..........** **..........** **..**

**Tottori/1/2007**  **..........** **..........** **..........** **..........** **..........** **..**

**Lincolnshire/1/2007**  **.....R....** **..........** **..........** **.........N** **..........** **..**

**California/1/2010**  **..........** **..........** **..........** **.........D** **..........** **..**

**Sweden/VIR165837/2011**  **..........** **..........** **..........** **.........D** **..........** **..**

**Kentucky/1/2011**  **..........** **..........** **..........** **.........D** **..........** **..**

**Kyonggi/SA1/2011**  **..........** **..........** **..........** **..........** **..........** **..**

**Dubai/1/2012**  **..........** **..........** **......R...** **.........D** **..........** **..**

**Rio Grande do Sul/1/2012** **..........** **..........** **......R...** **.........D** **..........** **..**

**Tennessee/28B/2014**  **..........** **..........** **..........** **......S..D** **..........** **..**

**Montana/9564-1/2015**  **..........** **..........** **..........** **......S..D** **..........** **..**

**Malaysia/1/2015**  **..........** **..........** **..........** **......S..D** **..........** **..**

**Georgia/121362-16/2016**  **..........** **..........** **..........** **......S..D** **..........** **..**

# **Figure S1.6 HA Alignment**

***10 20 30 40 50 60 70 80 90 100***

***....|....| ....|....| ....|....| ....|....| ....|....| ....|....| ....|....| ....|....| ....|....| ....|....|***

**South Africa/4/2003**  **SQNPISGNNT** **ATLCLGHHAV** **ANGTLVKTIS** **DDQIEVTNAT** **ELVQSISMGK** **ICNNSYRILD** **GRNCTLIDAM** **LGDPHCDAFQ** **YENWDLFIER** **SSAFSNCYPY**

**Ohio/1/2003**  **..........** **..........** **..........** **..........** **..........** **..........** **..........** **..........** **..........** **..........**

**Ohio/113461-3/2005**  **..........** **..........** **..........** **..........** **..........** **..........** **..........** **..........** **..........** **..........**

**Tottori/1/2007**  **..........** **..........** **..........** **.G........** **..........** **..........** **..........** **..........** **..........** **..........**

**Lincolnshire/1/2007**  **..........** **..........** **..........** **..........** **..........** **..........** **.K........** **..........** **..........** **..........**

**California/1/2010**  **......D...** **..........** **..........** **N.........** **..........** **..........** **.K........** **..........** **..........** **..........**

**Sweden/VIR165837/2011**  **....L.D...** **..........** **..........** **..........** **..........** **..........** **.K........** **..........** **..........** **..........**

**Kentucky/1/2011**  **......D...** **..........** **..........** **..........** **..........** **..........** **.K........** **..........** **..........** **..........**

**Kyonggi/SA1/2011**  **..........** **....M.....** **..........** **..........** **..........** **..........** **..........** **..........** **..........** **..........**

**Dubai/1/2012**  **......D...** **..........** **..........** **..........** **..........** **..........** **.K........** **..........** **..........** **..........**

**Rio Grande do Sul/1/2012** **......D...** **..........** **..........** **..........** **..........** **..........** **.K........** **..........** **..........** **..........**

**Tennessee/28B/2014**  **......D...** **..........** **..........** **..........** **......P...** **..........** **.K........** **..........** **..........** **..........**

**Montana/9564-1/2015**  **......D...** **..........** **..........** **..........** **......P...** **..........** **.K........** **..........** **..........** **..........**

**Malaysia/1/2015**  **.....ND...** **..........** **..........** **..........** **......P...** **..........** **.K........** **..........** **..........** **..........**

**Georgia/121362-16/2016**  **.....ND...** **..........** **....F.....** **..........** **......P...** **..........** **.K........** **..........** **..D.......** **..........**

***110 120 130 140 150 160 170 180 190 200***

***....|....| ....|....| ....|....| ....|....| ....|....| ....|....| ....|....| ....|....| ....|....| ....|....|***

**South Africa/4/2003**  **DIPDYASLRS** **IVASSGTLEF** **TAEGFTWTGV** **TQNGRSGACK** **RGSADSFFSR** **LNWLTKSGSS** **YPTLNVTMPN** **NKNFDKLYIW** **GIHHPSSNQE** **QTKLYIQESG**

**Ohio/1/2003**  **..........** **..........** **..........** **..........** **..........** **..........** **..........** **..........** **..........** **..........**

**Ohio/113461-3/2005**  **..........** **..........** **..........** **..........** **..........** **..........** **..........** **..........** **..........** **..........**

**Tottori/1/2007**  **..........** **..........** **..........** **..........** **..........** **..........** **.S........** **..........** **........K.** **..........**

**Lincolnshire/1/2007**  **...N......** **..........** **..........** **.......S..** **..........** **..........** **..........** **..........** **..........** **..........**

**California/1/2010**  **...N......** **..........** **..........** **.......S..** **..........** **..........** **..I.......** **..........** **..........** **..........**

**Sweden/VIR165837/2011**  **...N......** **..........** **..........** **.......S..** **..........** **..........** **..........** **..........** **..........** **......P...**

**Kentucky/1/2011**  **...N......** **..........** **..........** **.......S..** **..........** **..........** **..........** **..........** **..........** **..........**

**Kyonggi/SA1/2011**  **..........** **..........** **..........** **..........** **..........** **..........** **.S........** **..........** **........E.** **..........**

**Dubai/1/2012**  **...N......** **..........** **..........** **.......S..** **..........** **..........** **..........** **..........** **..........** **..........**

**Rio Grande do Sul/1/2012** **...N......** **..........** **..........** **.......S..** **..........** **..........** **..........** **..........** **..........** **..........**

**Tennessee/28B/2014**  **...N......** **..........** **..........** **.......S..** **..........** **..........** **..........** **..........** **.......T..** **..........**

**Montana/9564-1/2015**  **...N......** **..........** **..........** **....G..S..** **..........** **..........** **..........** **..........** **.......T..** **..........**

**Malaysia/1/2015**  **...N......** **..........** **..........** **.......S..** **..........** **..........** **..........** **..........** **.......T..** **..........**

**Georgia/121362-16/2016**  **...N......** **..........** **..........** **.......S..** **..........** **..........** **..........** **..........** **.......T..** **..........**

***210 220 230 240 250 260 270 280 290 300***

***....|....| ....|....| ....|....| ....|....| ....|....| ....|....| ....|....| ....|....| ....|....| ....|....|***

**South Africa/4/2003**  **RVTVSTKRSQ** **QTIIPNIGSR** **PWVRGQSGRI** **SIYWTIVKPG** **DILMINSNGN** **LVAPRGYFKL** **KTGKSSVMRS** **DVPIDICVSE** **CITPNGSISN** **DKPFQNVNKV**

**Ohio/1/2003**  **..........** **..........** **..........** **..........** **..........** **..........** **..........** **..........** **..........** **..........**

**Ohio/113461-3/2005**  **..........** **..........** **..........** **..........** **..........** **..........** **..........** **..........** **..........** **..........**

**Tottori/1/2007**  **..........** **..........** **..........** **..........** **..........** **..........** **..........** **..........** **..........** **..........**

**Lincolnshire/1/2007**  **..........** **..........** **..........** **..........** **..........** **..........** **..........** **..........** **..........** **..........**

**California/1/2010**  **..........** **..........** **..I......V** **..........** **..........** **..........** **..........** **..........** **..........** **..........**

**Sweden/VIR165837/2011**  **..........** **..........** **..........** **........S.** **..........** **..........** **..........** **..........** **..........** **..........**

**Kentucky/1/2011**  **..........** **..........** **..I.......** **..........** **..........** **..........** **..........** **..........** **..........** **..........**

**Kyonggi/SA1/2011**  **..........** **..........** **..........** **..........** **..........** **..........** **..........** **..........** **..........** **..........**

**Dubai/1/2012**  **..........** **..........** **..I.......** **..........** **..........** **..........** **..........** **..........** **..........** **..........**

**Rio Grande do Sul/1/2012** **..........** **..........** **..I.......** **..........** **..........** **..........** **..........** **..........** **..........** **..........**

**Tennessee/28B/2014**  **..........** **..........** **..I.......** **..........** **..........** **..........** **..........** **..........** **..........** **..........**

**Montana/9564-1/2015**  **..........** **..........** **..I.......** **..........** **..........** **..........** **..........** **..........** **..........** **..........**

**Malaysia/1/2015**  **..........** **..........** **..I.......** **..........** **..........** **..........** **..........** **..........** **..........** **..........**

**Georgia/121362-16/2016**  **..........** **..........** **..I.......** **..........** **..........** **..........** **..........** **..........** **..........** **..........**

***310 320 330 340 350 360 370 380 390 400***

***....|....| ....|....| ....|....| ....|....| ....|....| ....|....| ....|....| ....|....| ....|....| ....|....|***

**South Africa/4/2003**  **TYGKCPKYIR** **QNTLKLATGM** **RNVPEKQIRG** **IFGAIAGFIE** **NGWEGMVDGW** **YGFRYQNSEG** **TGQAADLKST** **QAAIDQINGK** **LNRVIERTNE** **KFHQIEKEFS**

**Ohio/1/2003**  **..........** **..........** **..........** **..........** **..........** **..........** **..........** **..........** **..........** **..........**

**Ohio/113461-3/2005**  **..........** **..........** **..........** **..........** **..........** **..........** **..........** **..........** **..........** **..........**

**Tottori/1/2007**  **..........** **..........** **..........** **..........** **..........** **..........** **..........** **..........** **..........** **..........**

**Lincolnshire/1/2007**  **..........** **..........** **..........** **..........** **..........** **..........** **..........** **..........** **..........** **..........**

**California/1/2010**  **..........** **..........** **..........** **..........** **..........** **..........** **..........** **..........** **..........** **..........**

**Sweden/VIR165837/2011**  **..E.......** **..........** **..........** **..........** **..........** **..........** **..........** **..........** **..........** **..........**

**Kentucky/1/2011**  **..........** **..........** **..........** **..........** **..........** **..........** **..........** **..........** **..........** **..........**

**Kyonggi/SA1/2011**  **..........** **..........** **..........** **..........** **..........** **..........** **..........** **..........** **..........** **..........**

**Dubai/1/2012**  **..........** **..........** **..........** **..........** **..........** **..........** **..........** **..........** **..........** **..........**

**Rio Grande do Sul/1/2012** **..........** **..........** **..........** **..........** **..........** **..........** **..........** **..........** **..........** **..........**

**Tennessee/28B/2014**  **..........** **..........** **..........** **..........** **..........** **..........** **..........** **..........** **..........** **..........**

**Montana/9564-1/2015**  **..........** **..........** **..........** **..........** **..........** **..........** **..........** **...T......** **..........** **..........**

**Malaysia/1/2015**  **..........** **..........** **..........** **..........** **..........** **..........** **..........** **..........** **..........** **..........**

**Georgia/121362-16/2016**  **..........** **..........** **..........** **..........** **..........** **..........** **..........** **..........** **..........** **..........**

***410 420 430 440 450 460 470 480 490 500***

***....|....| ....|....| ....|....| ....|....| ....|....| ....|....| ....|....| ....|....| ....|....| ....|....|***

**South Africa/4/2003**  **EVEGRIQDLE** **KYVEDTKIDL** **WSYNAELLVA** **LENQHTIDLT** **DAEMNKLFEK** **TRRQLRENAE** **DMGGGCFKIY** **HKCDNACIGS** **IRNGTYDHYI** **YRDEALNNRF**

**Ohio/1/2003**  **..........** **..........** **..........** **..........** **..........** **..........** **..........** **..........** **..........** **..........**

**Ohio/113461-3/2005**  **..........** **..........** **..........** **..........** **..........** **..........** **..........** **..........** **..........** **..........**

**Tottori/1/2007**  **..........** **..........** **..........** **..........** **...I......** **..........** **..........** **..........** **..........** **..........**

**Lincolnshire/1/2007**  **..........** **..........** **..........** **..........** **..........** **..........** **..........** **..........** **..........** **..........**

**California/1/2010**  **..........** **..........** **..........** **..........** **..........** **..........** **..........** **..........** **..........** **..........**

**Sweden/VIR165837/2011**  **..........** **..........** **..........** **..........** **..........** **..........** **..........** **..........** **..........** **..........**

**Kentucky/1/2011**  **..........** **..........** **..........** **..........** **..........** **..........** **..........** **..........** **..........** **..........**

**Kyonggi/SA1/2011**  **...E......** **..........** **..........** **..........** **..........** **..........** **..........** **..........** **..........** **..........**

**Dubai/1/2012**  **..........** **..........** **..........** **..........** **..........** **..........** **..........** **..........** **..........** **..........**

**Rio Grande do Sul/1/2012** **..........** **..........** **..........** **..........** **..........** **..........** **..........** **..........** **..........** **..........**

**Tennessee/28B/2014**  **..........** **..........** **..........** **..........** **..........** **..........** **..........** **...N......** **..........** **..........**

**Montana/9564-1/2015**  **...E......** **..........** **..........** **..........** **.........R** **..........** **..........** **...N......** **..........** **..........**

**Malaysia/1/2015 ..........** **..........** **..........** **..........** **..........** **..........** **..........** **...N......** **..........** **..........**

**Georgia/121362-16/2016**  **..........** **..........** **..........** **..........** **.........R** **...L......** **..........** **...N......** **..........** **..........**

***510 520 530 540 550***

***....|....| ....|....| ....|....| ....|....| ....|....|***

**South Africa/4/2003**  **QIKGVELKSG** **YKDWILWISF** **AISCFLICVV** **LLGFIMWACQ** **KGNIRCNICI**

**Ohio/1/2003**  **..........** **..........** **..........** **..........** **..........**

**Ohio/113461-3/2005**  **..........** **..........** **..........** **..........** **..........**

**Tottori/1/2007**  **..........** **..........** **......V...** **..........** **..........**

**Lincolnshire/1/2007**  **..........** **..........** **......V...** **..........** **..........**

**California/1/2010**  **..........** **..........** **......V...** **..........** **..........**

**Sweden/VIR165837/2011**  **..........** **..........** **......V...** **..........** **..........**

**Kentucky/1/2011**  **..........** **..........** **......V...** **..........** **..........**

**Kyonggi/SA1/2011**  **..........** **..........** **......V...** **..........** **..........**

**Dubai/1/2012**  **..........** **..........** **......V...** **..........** **..........**

**Rio Grande do Sul/1/2012** **..........** **..........** **......V...** **..........** **..........**

**Tennessee/28B/2014**  **..........** **..........** **..........** **..........** **..........**

**Montana/9564-1/2015**  **..........** **..........** **..........** **..........** **..........**

**Malaysia/1/2015 ..........** **..........** **..........** **..........** **..........**

**Georgia/121362-16/2016**  **..........** **..........** **..........** **..........** **..........**

# **Figure S1.7 NP Alignment**

***10 20 30 40 50 60 70 80 90 100***

***....|....| ....|....| ....|....| ....|....| ....|....| ....|....| ....|....| ....|....| ....|....| ....|....|***

**South Africa/4/2003**  **MASQGTKRSY** **EQMETDGERQ** **NATEIRASVG** **RMVGGIGRFY** **VQMCTELKLN** **DHEGRLIQNS** **ITIERMVLSA** **FDERRNKYLE** **EHPSAGKDPK** **KTGGPIYRRK**

**Ohio/1/2003**  **..........** **..........** **..........** **..........** **..........** **..........** **..........** **..........** **..........** **..........**

**Ohio/113461-3/2005**  **..........** **..........** **..........** **..........** **..........** **..........** **..........** **..........** **..........** **..........**

**Tottori/1/2007**  **..........** **..........** **..........** **..........** **..........** **..........** **..........** **..........** **..........** **..........**

**Lincolnshire/1/2007**  **..........** **..........** **..........** **..........** **..........** **..........** **..........** **..........** **..........** **..........**

**California/1/2010**  **..........** **..........** **..........** **..........** **..........** **..........** **..........** **..........** **..........** **..........**

**Sweden/VIR165837/2011**  **...H......** **..........** **..........** **..........** **..........** **..........** **..........** **..........** **..........** **..........**

**Kentucky/1/2011**  **..........** **..........** **..........** **..........** **..........** **..........** **..........** **..........** **..........** **..........**

**Kyonggi/SA1/2011**  **..........** **..........** **..........** **..........** **..........** **..........** **..........** **..........** **..........** **..........**

**Dubai/1/2012**  **..........** **..........** **..........** **..........** **..........** **..........** **..........** **..........** **..........** **..........**

**Rio Grande do Sul/1/2012** **..........** **..........** **..........** **..........** **..........** **..........** **..........** **..........** **..........** **..........**

**Tennessee/28B/2014**  **..........** **..........** **..........** **..........** **..........** **..........** **..........** **..........** **..........** **..........**

**Montana/9564-1/2015**  **..........** **..........** **..........** **..........** **..........** **..........** **..........** **..........** **..........** **..........**

**Malaysia/1/2015 ..........** **..........** **..........** **..........** **..........** **..........** **..........** **..........** **..........** **..........**

**Georgia/121362-16/2016**  **..........** **..........** **..........** **..........** **..........** **..........** **..........** **..........** **..........** **..........**

***110 120 130 140 150 160 170 180 190 200***

***....|....| ....|....| ....|....| ....|....| ....|....| ....|....| ....|....| ....|....| ....|....| ....|....|***

**South Africa/4/2003**  **DGKWMRELIL** **HDKEEIMRIW** **RQANNGEDAT** **AGLTHMMIWH** **SNLNDTTYQR** **TRALVRTGMD** **PRMCSLMQGS** **TLPRRSGAAG** **AAVKGVGTMV** **MELIRMIKRG**

**Ohio/1/2003**  **..........** **..........** **..........** **..........** **..........** **..........** **..........** **..........** **..........** **..........**

**Ohio/113461-3/2005**  **..........** **..........** **..........** **..........** **..........** **..........** **..........** **..........** **..........** **..........**

**Tottori/1/2007**  **..........** **..........** **..........** **..........** **..........** **..........** **..........** **..........** **..I.......** **..........**

**Lincolnshire/1/2007**  **..........** **..........** **..........** **..........** **..........** **..........** **..........** **..........** **..........** **..........**

**California/1/2010**  **..........** **..........** **..........** **..........** **..........** **..........** **..........** **..........** **..........** **..........**

**Sweden/VIR165837/2011**  **..........** **..........** **..........** **..........** **..........** **..........** **..........** **..........** **..........** **..........**

**Kentucky/1/2011**  **..........** **..........** **..........** **..........** **..........** **..........** **..........** **..........** **..........** **..........**

**Kyonggi/SA1/2011**  **..........** **Y.........** **..........** **..........** **..........** **..........** **..........** **..........** **..I.......** **..........**

**Dubai/1/2012**  **..........** **..........** **..........** **..........** **..........** **..........** **..........** **..........** **..........** **..........**

**Rio Grande do Sul/1/2012** **..........** **..........** **..........** **..........** **..........** **..........** **..........** **..........** **..........** **..........**

**Tennessee/28B/2014**  **..........** **..........** **..........** **..........** **..........** **..........** **..........** **..........** **..........** **..........**

**Montana/9564-1/2015**  **..........** **..........** **..........** **..........** **..........** **..........** **..........** **..........** **..........** **..........**

**Malaysia/1/2015 ..........** **..........** **..........** **..........** **..........** **..........** **..........** **..........** **..........** **..........**

**Georgia/121362-16/2016**  **..........** **..........** **..........** **..........** **..........** **..........** **..........** **..........** **..........** **..........**

***210 220 230 240 250 260 270 280 290 300***

***....|....| ....|....| ....|....| ....|....| ....|....| ....|....| ....|....| ....|....| ....|....| ....|....|***

**South Africa/4/2003**  **INDRNFWRGE** **NGRKTRIAYE** **RMCNILKGKF** **QTAAQRAMMD** **QVREGRNPGN** **AEIEDLIFLA** **RSALILRGSV** **AHKSCLPACV** **YGLAVTSGYD** **FEKEGYSLVG**

**Ohio/1/2003**  **..........** **...R......** **..........** **..........** **..........** **..........** **..........** **..........** **..........** **..........**

**Ohio/113461-3/2005**  **..........** **...R......** **..........** **..........** **..........** **..........** **..........** **..........** **..........** **..........**

**Tottori/1/2007**  **..........** **...R......** **..........** **..........** **..........** **..........** **..........** **..........** **..........** **..........**

**Lincolnshire/1/2007**  **..........** **...R......** **..........** **..........** **..........** **..........** **..........** **..........** **..........** **..........**

**California/1/2010**  **..........** **...R......** **..........** **..........** **..........** **..........** **..........** **..........** **..........** **..........**

**Sweden/VIR165837/2011**  **..........** **...R......** **..........** **..........** **..........** **..........** **..........** **..........** **..........** **..........**

**Kentucky/1/2011**  **..........** **...R......** **..........** **..........** **..........** **..........** **..........** **..........** **..........** **..........**

**Kyonggi/SA1/2011**  **..........** **...R......** **..........** **..........** **..........** **..........** **..........** **..........** **..........** **..........**

**Dubai/1/2012**  **..........** **...R......** **..........** **..........** **..........** **..........** **..........** **..........** **..........** **..........**

**Rio Grande do Sul/1/2012** **..........** **...R......** **..........** **..........** **..........** **..........** **..........** **..........** **..........** **..........**

**Tennessee/28B/2014**  **..........** **...R......** **..........** **..........** **..........** **..........** **..........** **..........** **..........** **..........**

**Montana/9564-1/2015**  **..........** **...R......** **..........** **..........** **..........** **..........** **..........** **..........** **..........** **..........**

**Malaysia/1/2015**  **..........** **...R......** **..........** **..........** **..........** **..........** **..........** **..........** **..........** **..........**

**Georgia/121362-16/2016**  **..........** **...R......** **..........** **..........** **..........** **..........** **..........** **..........** **..........** **..........**

***310 320 330 340 350 360 370 380 390 400***

***....|....| ....|....| ....|....| ....|....| ....|....| ....|....| ....|....| ....|....| ....|....| ....|....|***

**South Africa/4/2003**  **IDPFKLLQNS** **QIFSLIRPKE** **NPAHKSQLVW** **MACHSAAFED** **LRVLNFIRGT** **KVIPRGQLTT** **RGVQIASNEN** **METIDSSTLE** **LRSKYWAIRT** **RSGGNTSQQR**

**Ohio/1/2003**  **..........** **..........** **..........** **..........** **..........** **..........** **..........** **..........** **..........** **..........**

**Ohio/113461-3/2005**  **..........** **..........** **..........** **..........** **..........** **..........** **..........** **..........** **..........** **..........**

**Tottori/1/2007**  **..........** **..........** **..........** **..........** **M.........** **..........** **..........** **..........** **..........** **..........**

**Lincolnshire/1/2007**  **..........** **..........** **..........** **..........** **..........** **..........** **..........** **..........** **..........** **..........**

**California/1/2010**  **..........** **..........** **..........** **..........** **..........** **..........** **..........** **..........** **..........** **..........**

**Sweden/VIR165837/2011**  **..........** **..........** **..........** **..........** **..........** **..........** **..........** **..........** **..........** **.........K**

**Kentucky/1/2011**  **..........** **..........** **..........** **..........** **..........** **..........** **..........** **..........** **..........** **..........**

**Kyonggi/SA1/2011**  **..........** **..........** **..........** **..........** **M.........** **..........** **..........** **......C...** **..........** **..........**

**Dubai/1/2012**  **..........** **..........** **..........** **..........** **..........** **..........** **..........** **..........** **..........** **..........**

**Rio Grande do Sul/1/2012** **..........** **..........** **..........** **..........** **..........** **..........** **..........** **..........** **..........** **..........**

**Tennessee/28B/2014**  **..........** **..........** **..........** **..........** **..........** **..........** **..........** **..........** **..........** **..........**

**Montana/9564-1/2015**  **..........** **..........** **..........** **..........** **..........** **..........** **..........** **..........** **..........** **..........**

**Malaysia/1/2015**  **..........** **..........** **..........** **..........** **..........** **..........** **..........** **..........** **..........** **..........**

**Georgia/121362-16/2016**  **..........** **..........** **..........** **..........** **..........** **..........** **..........** **..........** **..........** **..........**

***410 420 430 440 450 460 470 480 490***

***....|....| ....|....| ....|....| ....|....| ....|....| ....|....| ....|....| ....|....| ....|....| ....|...***

**South Africa/4/2003**  **ASAGQISVQP** **TFSVQRNLPF** **ERATIMAAFT** **GNTEGRTSDM** **RTEIIRMMEN** **AKSEDVSFQG** **RGVFELSDEK** **ATNPIVPSFD** **MSNEGSYFFG** **DNAEEFDS**

**Ohio/1/2003**  **..........** **..........** **..........** **..........** **..........** **..........** **..........** **..........** **..........** **........**

**Ohio/113461-3/2005**  **..........** **..........** **..........** **..........** **..........** **..........** **..........** **..........** **..........** **........**

**Tottori/1/2007**  **..........** **..........** **..........** **..........** **..........** **..........** **..........** **..........** **..........** **........**

**Lincolnshire/1/2007**  **..........** **..........** **..........** **..........** **..........** **..........** **..........** **..........** **..........** **........**

**California/1/2010**  **..........** **..........** **..........** **..........** **..........** **..........** **..........** **..........** **..........** **........**

**Sweden/VIR165837/2011**  **..........** **..........** **..........** **..........** **..........** **..........** **..........** **..........** **..........** **.**

**Kentucky/1/2011**  **..........** **..........** **..........** **..........** **..........** **..........** **..........** **..........** **..........** **........**

**Kyonggi/SA1/2011**  **..........** **..........** **..........** **..........** **..........** **..........** **..........** **..........** **..........** **........**

**Dubai/1/2012**  **..........** **..........** **..........** **..........** **..........** **..........** **..........** **..........** **..........** **........**

**Rio Grande do Sul/1/2012** **..........** **..........** **..........** **..........** **..........** **..........** **..........** **..........** **..........** **........**

**Tennessee/28B/2014**  **..........** **..........** **.........I** **..........** **..........** **..........** **..........** **..........** **..........** **........**

**Montana/9564-1/2015**  **..........** **..........** **.........I** **..........** **..........** **..........** **..........** **..........** **..........** **........**

**Malaysia/1/2015 ..........** **..........** **.........I** **..........** **..........** **..........** **..........** **..........** **..........** **........**

**Georgia/121362-16/2016**  **..........** **..........** **.........I** **..........** **..........** **..........** **..........** **..........** **..........** **........**

# **Figure S1.8 NA Alignment**

***10 20 30 40 50 60 70 80 90 100***

***....|....| ....|....| ....|....| ....|....| ....|....| ....|....| ....|....| ....|....| ....|....| ....|....|***

**South Africa/4/2003**  **MNPNQKIIAI** **GFASLGILII** **NVILHVVSII** **VTVLVLNNNR** **TDLNCKGTII** **REYNETVRVE** **KITQWYNTST** **IKYIERPSNE** **YYMNNTEPLC** **EAQGFAPFSK**

**Ohio/1/2003**  **..........** **..........** **..........** **..........** **..........** **..........** **..........** **..........** **..........** **..........**

**Ohio/113461-3/2005**  **.......M..** **..........** **..........** **....A.....** **..........** **..........** **..........** **..........** **..........** **..........**

**Tottori/1/2007**  **.......M..** **..........** **..........** **....A.....** **..........** **K.........** **..........** **..........** **..........** **..........**

**Lincolnshire/1/2007**  **..........** **..........** **..........** **....A....K** **..........** **..........** **.........A** **..........** **..........** **..........**

**California/1/2010**  **.......M..** **..........** **..........** **....A.....** **..........** **..........** **..........** **.......P..** **..........** **..........**

**Sweden/VIR165837/2011**  **.......M..** **..........** **..F.......** **....A..C..** **..........** **..........** **..........** **..........** **......D...** **..........**

**Kentucky/1/2011**  **.......M..** **..........** **S......N..** **....A.....** **..........** **..........** **..........** **..........** **..........** **..........**

**Kyonggi/SA1/2011**  **....P..M..** **..........** **..........** **....T.....** **..........** **K.........** **..........** **..........** **..........** **..........**

**Dubai/1/2012**  **.......M..** **..........** **..........** **....A.....** **..........** **..........** **..........** **..........** **..........** **..........**

**Rio Grande do Sul/1/2012** **.......M..** **..........** **..........** **....A.....** **..........** **..........** **..........** **..........** **..........** **..........**

**Tennessee/28B/2014**  **..........** **..........** **..........** **....A.....** **..........** **..........** **..........** **..........** **..........** **..........**

**Montana/9564-1/2015**  **..........** **..........** **..........** **....A.....** **..........** **..........** **..........** **..........** **..........** **..........**

**Malaysia/1/2015 ..........** **..........** **..........** **....A.....** **..........** **..........** **..........** **..........** **..........** **..........**

**Georgia/121362-16/2016**  **..........** **..........** **..........** **....A.....** **......E...** **..........** **..........** **.....K....** **..........** **..........**

***110 120 130 140 150 160 170 180 190 200***

***....|....| ....|....| ....|....| ....|....| ....|....| ....|....| ....|....| ....|....| ....|....| ....|....|***

**South Africa/4/2003**  **DNGIRIGSRG** **HVFVIREPFV** **SCSPSECRTF** **FLTQGSLLND** **KHSNGTVKDR** **SPYRTLMSVK** **IGQSPNVYQA** **RFESVAWSAT** **ACHDGKKWMT** **VGVTGPDNQA**

**Ohio/1/2003**  **..........** **..........** **..........** **..........** **..........** **..........** **..........** **..........** **..........** **..........**

**Ohio/113461-3/2005**  **..........** **..........** **..........** **..........** **..........** **..........** **..........** **..........** **.....R....** **..........**

**Tottori/1/2007**  **..........** **..........** **..........** **..........** **......I...** **..........** **..........** **..........** **..........** **..........**

**Lincolnshire/1/2007**  **..........** **..........** **..........** **..........** **..........** **..........** **..........** **..........** **..........** **..........**

**California/1/2010**  **..........** **..........** **..........** **..........** **..........** **..........** **..........** **..........** **..........** **..........**

**Sweden/VIR165837/2011**  **.C........** **..........** **..........** **..........** **..L.......** **..........** **..........** **..........** **..........** **..........**

**Kentucky/1/2011**  **..........** **..........** **..........** **..........** **..........** **..........** **..........** **..........** **..........** **..........**

**Kyonggi/SA1/2011**  **..........** **..........** **..........** **..........** **......I...** **..........** **..........** **..........** **..........** **..........**

**Dubai/1/2012**  **..........** **..........** **..........** **..........** **..........** **..........** **..........** **..........** **..........** **..........**

**Rio Grande do Sul/1/2012** **..........** **..........** **..........** **..........** **..........** **..........** **........H.** **..........** **..........** **..........**

**Tennessee/28B/2014**  **..........** **..........** **..........** **..........** **..........** **..........** **..........** **..........** **..........** **..........**

**Montana/9564-1/2015**  **..........** **..........** **..........** **..........** **..........** **..........** **..........** **..........** **..........** **..........**

**Malaysia/1/2015 ..........** **..........** **..........** **..........** **..........** **..........** **..........** **..........** **..........** **..........**

**Georgia/121362-16/2016**  **..........** **..........** **..........** **..........** **..........** **..........** **..........** **..........** **..........** **..........**

***210 220 230 240 250 260 270 280 290 300***

***....|....| ....|....| ....|....| ....|....| ....|....| ....|....| ....|....| ....|....| ....|....| ....|....|***

**South Africa/4/2003**  **IAVVNYGGVP** **VDIINSWAGD** **ILRTQESSCT** **CIKGDCYWVM** **TDGPANRQAK** **YRIFKAKDGR** **VIGQTDISFN** **GGHIEECSCY** **PNEGKVECIC** **RDNWTGTNRP**

**Ohio/1/2003**  **..........** **..........** **..........** **..........** **..........** **..........** **..........** **E.........** **..........** **..........**

**Ohio/113461-3/2005**  **..........** **..........** **..........** **..........** **..........** **..........** **..........** **..........** **..........** **..........**

**Tottori/1/2007**  **..........** **.........N** **..........** **..........** **..........** **..........** **.......N..** **..........** **..........** **..........**

**Lincolnshire/1/2007**  **..........** **..........** **..........** **..........** **..........** **..........** **..........** **..........** **..........** **..........**

**California/1/2010**  **..........** **..........** **..........** **..........** **..........** **.........K** **..........** **..........** **..........** **..........**

**Sweden/VIR165837/2011**  **..........** **..........** **..........** **..........** **..........** **.........K** **..........** **..........** **..........** **..........**

**Kentucky/1/2011**  **..........** **..........** **..........** **..........** **..........** **.........K** **..........** **..........** **..........** **..........**

**Kyonggi/SA1/2011**  **..........** **.........N** **..........** **..........** **..........** **..........** **.......N..** **..........** **..........** **..........**

**Dubai/1/2012**  **....S.....** **..........** **..........** **..........** **..........** **.........K** **..........** **..........** **..........** **..........**

**Rio Grande do Sul/1/2012** **....S.....** **..........** **..........** **..........** **..........** **.........K** **..........** **..........** **..........** **..........**

**Tennessee/28B/2014**  **..........** **..........** **..........** **..........** **..........** **.K.....N.K** **..........** **..........** **..........** **..........**

**Montana/9564-1/2015**  **..........** **..........** **..........** **..........** **..........** **.K.....N.K** **.....N....** **..........** **..........** **..........**

**Malaysia/1/2015 ..........** **..........** **..........** **..........** **..........** **.K.....N.K** **.....N....** **..........** **..........** **..........**

**Georgia/121362-16/2016**  **..........** **...V......** **..........** **..........** **..........** **.K.....N.K** **..........** **..........** **..........** **..........**

***310 320 330 340 350 360 370 380 390 400***

***....|....| ....|....| ....|....| ....|....| ....|....| ....|....| ....|....| ....|....| ....|....| ....|....|***

**South Africa/4/2003**  **ILVISSDLSY** **TVGYLCAGIP** **TDTPRGEDSQ** **FTGSCTSPLG** **NKGYGVKGFG** **FRQGTDVWAG** **RTISRTSRSG** **FEIIKIRNGW** **TQNSKDQIRR** **QVIIDDPNWS**

**Ohio/1/2003**  **..........** **..........** **..........** **..........** **..........** **..........** **..........** **..........** **..........** **..........**

**Ohio/113461-3/2005**  **..........** **..........** **..........** **......N...** **..........** **..........** **..........** **..........** **..........** **..........**

**Tottori/1/2007**  **..........** **..........** **..........** **......N...** **..........** **..........** **..........** **..........** **..........** **..........**

**Lincolnshire/1/2007**  **..........** **..........** **..........** **......N...** **..........** **..........** **..........** **..........** **..........** **..........**

**California/1/2010**  **..........** **..........** **..........** **......N...** **..........** **..........** **..........** **..........** **..........** **..........**

**Sweden/VIR165837/2011**  **..........** **...F.W....** **.A.H......** **..A...N...** **.......S..** **.L........** **....K..I.R** **..........** **..........** **..........**

**Kentucky/1/2011**  **..........** **..........** **..........** **......N...** **..........** **..........** **..........** **..........** **..........** **..........**

**Kyonggi/SA1/2011**  **..........** **..........** **..........** **......N...** **..........** **..........** **..........** **..........** **..........** **..........**

**Dubai/1/2012**  **..........** **..........** **..........** **......N...** **..........** **..........** **..........** **..........** **..........** **..........**

**Rio Grande do Sul/1/2012** **..........** **..........** **..........** **......N...** **..........** **..........** **..........** **..........** **..........** **..........**

**Tennessee/28B/2014**  **..........** **..........** **..........** **......N...** **..........** **..........** **..........** **..........** **..........** **..........**

**Montana/9564-1/2015**  **..........** **..........** **..........** **......N...** **..........** **..........** **..........** **..........** **..........** **..........**

**Malaysia/1/2015 ..........** **..........** **..........** **......N...** **..........** **..........** **..........** **..........** **..........** **..........**

**Georgia/121362-16/2016**  **..........** **..........** **..........** **......N...** **..........** **..........** **..........** **..........** **..........** **..........**

***410 420 430 440 450 460 470***

***....|....| ....|....| ....|....| ....|....| ....|....| ....|....| ....|....|***

**South Africa/4/2003**  **GYSGSFTLPV** **ELTKKGCLVP** **CFWVEMIRGK** **PEETTIWTSS** **SSIVMCGVDH** **KIASWSWHDG** **AILPFDIDKM**

**Ohio/1/2003**  **..........** **..........** **..........** **..........** **..........** **..........** **.........**

**Ohio/113461-3/2005**  **..........** **..........** **..........** **..........** **..........** **..........** **..........**

**Tottori/1/2007**  **..........** **..........** **..........** **..........** **..........** **..........** **..........**

**Lincolnshire/1/2007**  **..........** **.....E....** **..........** **..........** **..........** **..........** **..........**

**California/1/2010**  **..........** **.....E....** **..........** **..........** **..........** **..........** **..........**

**Sweden/VIR165837/2011**  **.NR.YI.I.K** **.I.I.EG.I.** **SLR..T....** **..........** **...M......** **.MPR......** **..........**

**Kentucky/1/2011**  **..........** **.....E....** **..........** **..........** **..........** **..........** **..........**

**Kyonggi/SA1/2011**  **..........** **..........** **..........** **..........** **..........** **..........** **..........**

**Dubai/1/2012**  **..........** **.....E....** **..........** **..........** **..........** **..........** **..........**

**Rio Grande do Sul/1/2012** **..........** **.....E....** **..........** **..........** **..........** **..........** **..........**

**Tennessee/28B/2014**  **..........** **.....E....** **..........** **...K......** **..........** **..........** **..........**

**Montana/9564-1/2015**  **..........** **.....E....** **..........** **...K......** **..........** **..........** **..........**

**Malaysia/1/2015 ..........** **.....E....** **..........** **...K......** **..........** **..........** **..........**

**Georgia/121362-16/2016**  **..........** **.....E....** **..........** **...K......** **..........** **..........** **..........**

# **Figure S1.9 MP1 Alignment**

***10 20 30 40 50 60 70 80 90 100***

***....|....| ....|....| ....|....| ....|....| ....|....| ....|....| ....|....| ....|....| ....|....| ....|....|***

**South Africa/4/2003**  **MSLLTEVETY** **VLSIVPSGPL** **KAEIAQRLED** **VFAGKNTDLE** **ALMEWLKTRP** **ILSPLTKGIL** **GFVFTLTVPS** **ERGLQRRRFV** **QNALSGNGDP** **NNMDRAVKLY**

**Ohio/1/2003**  **..........** **..........** **..........** **..........** **..........** **..........** **..........** **..........** **..........** **..........**

**Ohio/113461-3/2005**  **..........** **..........** **..........** **..........** **..........** **..........** **..........** **..........** **..........** **..........**

**Tottori/1/2007**  **..........** **....I.....** **..........** **..........** **..........** **..........** **..........** **..........** **..........** **..........**

**Lincolnshire/1/2007**  **..........** **..........** **..........** **..........** **..........** **..........** **..........** **..........** **..........** **..........**

**California/1/2010**  **..........** **..........** **..........** **..........** **..........** **..........** **..........** **..........** **..........** **..........**

**Sweden/VIR165837/2011**  **..........** **....I.....** **..........** **..........** **..........** **..........** **..........** **..........** **..........** **..........**

**Kentucky/1/2011**  **..........** **....I.....** **..........** **..........** **..........** **..........** **..........** **..........** **..........** **..........**

**Kyonggi/SA1/2011**  **..........** **....I.....** **.....R....** **....R.A...** **..........** **..........** **..........** **..........** **..........** **..........**

**Dubai/1/2012**  **..........** **....I.....** **..........** **..........** **..........** **..........** **..........** **..........** **..........** **..........**

**Rio Grande do Sul/1/2012** **..........** **....I.....** **..........** **..........** **..........** **..........** **..........** **..........** **..........** **..........**

**Tennessee/28B/2014**  **..........** **....M.....** **..........** **..........** **..........** **..........** **..........** **..........** **..........** **..........**

**Montana/9564-1/2015**  **..........** **....M.....** **..........** **..........** **..........** **..........** **..........** **..........** **..........** **..........**

**Malaysia/1/2015 ..........** **....M.....** **..........** **..........** **..........** **..........** **..........** **..........** **..........** **..........**

**Georgia/121362-16/2016**  **..........** **....M.....** **..........** **..........** **..........** **..........** **..........** **..........** **..........** **..........**

***110 120 130 140 150 160 170 180 190 200***

***....|....| ....|....| ....|....| ....|....| ....|....| ....|....| ....|....| ....|....| ....|....| ....|....|***

**South Africa/4/2003**  **RKLKREITFH** **GAKEVALSYS** **TGALASCMGL** **IYNRMGTVTT** **EVAFGLVCAT** **CEQIADSQHR** **SHRQMVTTTN** **PLIRHENRMV** **LASTTAKAME** **QMAGSSEQAA**

**Ohio/1/2003**  **..........** **..........** **..........** **..........** **..........** **..........** **..........** **..........** **..........** **..........**

**Ohio/113461-3/2005**  **..........** **..........** **..........** **..........** **..........** **..........** **..........** **..........** **..........** **..........**

**Tottori/1/2007**  **..........** **..........** **..........** **..........** **..........** **..........** **..........** **..........** **..........** **..........**

**Lincolnshire/1/2007**  **..........** **..........** **..........** **..........** **..........** **..........** **..........** **..........** **..........** **..........**

**California/1/2010**  **..........** **..........** **..........** **..........** **..........** **..........** **..........** **..........** **..........** **..........**

**Sweden/VIR165837/2011**  **..........** **..........** **..........** **..........** **..........** **..........** **..........** **..........** **..........** **..........**

**Kentucky/1/2011**  **..........** **..........** **..........** **..........** **..........** **..........** **..........** **..........** **..........** **..........**

**Kyonggi/SA1/2011**  **..........** **..........** **..........** **..........** **..........** **..........** **..........** **..........** **..........** **..........**

**Dubai/1/2012**  **..........** **..........** **..........** **..........** **..........** **..........** **..........** **..........** **..........** **..........**

**Rio Grande do Sul/1/2012** **..........** **..........** **..........** **..........** **..........** **..........** **..........** **..........** **..........** **..........**

**Tennessee/28B/2014**  **..........** **..........** **..........** **..........** **..........** **..........** **..........** **..........** **..........** **..........**

**Montana/9564-1/2015**  **..........** **..........** **..........** **..........** **..........** **..........** **..........** **..........** **..........** **..........**

**Malaysia/1/2015 ..........** **..........** **..........** **..........** **..........** **..........** **..........** **..........** **..........** **..........**

**Georgia/121362-16/2016**  **..........** **..........** **..........** **..........** **..........** **..........** **..........** **..........** **..........** **..........**

***210 220 230 240 250***

***....|....| ....|....| ....|....| ....|....| ....|....| ..***

**South Africa/4/2003**  **EAMEVASRAR** **QMVQAMRTIG** **THPSSSAGLK** **DDLLENLQAY** **QKRMGVQMQR** **FK**

**Ohio/1/2003**  **..........** **..........** **..........** **..........** **..........** **..**

**Ohio/113461-3/2005**  **..........** **..........** **..........** **..........** **..........** **..**

**Tottori/1/2007**  **..........** **..........** **..........** **..........** **..........** **..**

**Lincolnshire/1/2007**  **..........** **..........** **..........** **..........** **..........** **..**

**California/1/2010**  **..........** **..........** **..........** **..........** **..........** **..**

**Sweden/VIR165837/2011**  **......N...** **..........** **..........** **..........** **..........** **..**

**Kentucky/1/2011**  **..........** **..........** **..........** **..........** **..........** **..**

**Kyonggi/SA1/2011**  **..........** **..........** **..........** **..........** **..........** **..**

**Dubai/1/2012**  **..........** **..........** **..........** **..........** **..........** **..**

**Rio Grande do Sul/1/2012** **..........** **..........** **..........** **..........** **..........** **..**

**Tennessee/28B/2014**  **..........** **..........** **..........** **..........** **..........** **..**

**Montana/9564-1/2015**  **..........** **..........** **..........** **..........** **..........** **..**

**Malaysia/1/2015 ..........** **..........** **..........** **..........** **..........** **..**

**Georgia/121362-16/2016**  **..........** **..........** **..........** **..........** **..........** **..**

# **Figure S1.10 MP2 Alignment**

***10 20 30 40 50 60 70 80 90***

***....|....| ....|....| ....|....| ....|....| ....|....| ....|....| ....|....| ....|....| ....|....| ....|..***

**South Africa/4/2003**  **MSLLTEVETP** **TRNGWECKCS** **DSSDPLVIAA** **SIIGILHLIL** **WILDRLFFKF** **IYRRLKYGLK** **RGPSTEGVPE** **SMREEYRQEQ** **QNAVDVDDGH** **FVNIELE**

**Ohio/1/2003**  **..........** **..........** **..........** **..........** **..........** **..........** **..........** **..........** **......E...** **.......**

**Ohio/113461-3/2005**  **..........** **..........** **..........** **..........** **..........** **........M.** **..........** **..........** **..........** **.......**

**Lincolnshire/1/2007**  **..........** **..........** **..........** **..........** **..........** **........M.** **..........** **..........** **..........** **......**

**Tottori/1/2007**  **..........** **..........** **..........** **..........** **..........** **..H.....M.** **..........** **..........** **..........** **.......**

**California/1/2010**  **..........** **..S.......** **..........** **..........** **..........** **........M.** **..........** **..........** **..........** **.......**

**Sweden/VIR165837/2011**  **..........** **..........** **..........** **..........** **..........** **........M.** **..........** **..........** **..........** **.....P.**

**Kentucky/1/2011**  **..........** **..........** **..........** **..........** **..........** **........M.** **..........** **.....F....** **..........** **.......**

**Kyonggi/SA1/2011**  **..........** **..........** **..........** **..........** **..........** **..H.....M.** **..........** **..........** **..........** **.......**

**Dubai/1/2012**  **..........** **..........** **..........** **..........** **..........** **........M.** **..........** **.....F....** **..........** **.......**

**Rio Grande do Sul/1/2012** **..........** **..........** **..........** **..........** **..........** **........M.** **..........** **.....F....** **..........** **.......**

**Tennessee/28B/2014**  **..........** **..........** **..........** **..........** **..........** **........M.** **..........** **..........** **..........** **.......**

**Montana/9564-1/2015**  **..........** **..........** **..........** **..........** **..........** **........M.** **..........** **......L...** **..........** **.......**

**Malaysia/1/2015 ..........** **..........** **..........** **..........** **..........** **........M.** **..........** **..........** **..........** **.......**

**Georgia/121362-16/2016**  **..........** **..........** **..........** **..........** **..........** **........M.** **..........** **..........** **..........** **.......**

# **Figure S1.11 NS1 Alignment**

***10 20 30 40 50 60 70 80 90 100***

***....|....| ....|....| ....|....| ....|....| ....|....| ....|....| ....|....| ....|....| ....|....| ....|....|***

**South Africa/4/2003**  **MDSNTVSSFQ** **VDCFLWHVRK** **RVADQELGDA** **PFLDRLRRDQ** **KSLRGRGSTL** **GLDIETATHA** **GKQIVEQILE** **KESDEALKMT** **IASVPTSRYL** **TDMTLDEMSR**

**Ohio/1/2003**  **..........** **..........** **.F........** **..........** **..........** **..........** **..........** **..........** **..........** **..........**

**Ohio/113461-3/2005**  **..........** **..........** **.F........** **..........** **..........** **..........** **..........** **..........** **..........** **..........**

**Tottori/1/2007**  **..........** **..........** **.F........** **..........** **..........** **..........** **..........** **..........** **..........** **.....G....**

**Lincolnshire/1/2007**  **..........** **..........** **.F........** **..........** **..........** **..........** **..........** **..........** **..........** **..........**

**California/1/2010**  **..........** **..........** **.F........** **..........** **..........** **..........** **.....K....** **..........** **..........** **..........**

**Sweden/VIR165837/2011**  **...T......** **..........** **.F........** **..........** **..........** **..........** **.....K....** **..........** **..........** **..........**

**Kentucky/1/2011**  **..........** **..........** **.F........** **..........** **..........** **..........** **.....K....** **..........** **..........** **..........**

**Dubai/1/2012**  **..........** **..........** **.F........** **..........** **..........** **..........** **.....K....** **..........** **..........** **..........**

**Rio Grande do Sul/1/2012** **..........** **..........** **.F........** **..........** **..........** **..........** **.....K....** **..........** **..........** **..........**

**Tennessee/28B/2014**  **..........** **..........** **.F........** **..........** **..........** **..........** **.....K....** **..........** **..........** **..........**

**Montana/9564-1/2015**  **..........** **..........** **.F........** **..........** **..........** **..........** **.....K....** **..........** **..........** **..........**

**Malaysia/1/2015 ..........** **..........** **.F........** **..........** **..........** **..........** **.....K....** **..........** **..........** **..........**

**Georgia/121362-16/2016**  **..........** **..........** **.F........** **..........** **..........** **..........** **.....K....** **..........** **..........** **..........**

***110 120 130 140 150 160 170 180 190 200***

***....|....| ....|....| ....|....| ....|....| ....|....| ....|....| ....|....| ....|....| ....|....| ....|....|***

**South Africa/4/2003**  **DWFMLMPKQK** **VTGSLCIRMD** **QAIMDKNITL** **KANFSVIFER** **LETLILLRAF** **TEEGAIVGEI** **SPLPSLPGHT** **NEDVKNAIGV** **LIGGLKWNDN** **TVRISETLQR**

**Ohio/1/2003**  **..........** **..........** **........I.** **..........** **..........** **.....V....** **..........** **..........** **..........** **..........**

**Ohio/113461-3/2005**  **..........** **..........** **........I.** **..........** **..........** **.....V....** **..........** **..........** **..........** **..........**

**Tottori/1/2007**  **..........** **..........** **........I.** **..........** **..........** **.....V....** **..........** **..........** **..........** **..........**

**Lincolnshire/1/2007**  **..........** **..........** **........I.** **..........** **..........** **.....V....** **..........** **..........** **..........** **..........**

**California/1/2010**  **..........** **..........** **........I.** **..........** **..........** **.....V....** **..........** **..........** **..........** **..........**

**Sweden/VIR165837/2011**  **..........** **..........** **........I.** **..........** **..........** **.....V....** **..........** **..........** **..........** **..........**

**Kentucky/1/2011**  **..........** **..........** **........I.** **..........** **..........** **.....V....** **..........** **..........** **..........** **..........**

**Dubai/1/2012**  **..........** **..........** **........I.** **..........** **..........** **.....V....** **..........** **..........** **..........** **..........**

**Rio Grande do Sul/1/2012** **..........** **..........** **........I.** **..........** **..........** **.....V....** **..........** **..........** **..........** **..........**

**Tennessee/28B/2014**  **..........** **..........** **........I.** **..........** **..........** **.....V....** **..........** **..........** **..........** **..........**

**Montana/9564-1/2015**  **..........** **..........** **........I.** **..........** **..........** **.....V....** **..........** **..........** **..........** **..........**

**Malaysia/1/2015 ..........** **..........** **........I.** **..........** **..........** **.....V....** **..........** **..........** **..........** **..........**

**Georgia/121362-16/2016**  **..........** **..........** **........I.** **..........** **..........** **.....V....** **..........** **..........** **..........** **..........**

***210***

***....|....| ....|....***

**South Africa/4/2003**  **FAWRSSHENG** **RPSFPSKQK**

**Ohio/1/2003**  **..........** **.........**

**Ohio/113461-3/2005**  **..........** **.........**

**Tottori/1/2007**  **..........** **.........**

**Lincolnshire/1/2007**  **.........R** **.........**

**California/1/2010**  **........I.** **.........**

**Sweden/VIR165837/2011**  **......N.I.** **.........**

**Kentucky/1/2011**  **........I.** **.........**

**Dubai/1/2012**  **........I.** **.........**

**Rio Grande do Sul/1/2012** **........I.** **.........**

**Tennessee/28B/2014**  **......N.I.** **.S.......**

**Montana/9564-1/2015**  **......N.I.** **.S.......**

**Malaysia/1/2015 ......N.I.** **.S.......**

**Georgia/121362-16/2016**  **......N.I.** **.S.......**

# **Figure S1.12 NEP Alignment**

***10 20 30 40 50 60 70 80 90 100***

***....|....| ....|....| ....|....| ....|....| ....|....| ....|....| ....|....| ....|....| ....|....| ....|....|***

**South Africa/4/2003**  **MDSNTVSSFQ** **DILMRMSKMQ** **LGSSSEDLNG** **MIIRLESLKL** **YRDSLGEAVM** **RMGDLHSLQS** **RNEKWREQLS** **QKFEEIRWLI** **EEVRHRLKNT** **ENSFEQITFM**

**Ohio/1/2003**  **..........** **..........** **..........** **..........** **..........** **..........** **..........** **..........** **..........** **..........**

**Ohio/113461-3/2005**  **..........** **..........** **..........** **..........** **..........** **..........** **..........** **..........** **..........** **..........**

**Tottori/1/2007**  **..........** **..........** **..........** **..........** **..........** **..........** **..........** **.....V....** **..........** **..........**

**Lincolnshire/1/2007**  **..........** **..........** **..........** **..........** **..........** **.I........** **..........** **..........** **..........** **..........**

**California/1/2010**  **..........** **..........** **..........** **..........** **..........** **.L........** **..........** **..........** **..........** **..........**

**Sweden/VIR165837/2011**  **...T......** **..........** **..........** **..........** **..........** **.L........** **..........** **..........** **..........** **..........**

**Kentucky/1/2011**  **..........** **..........** **..........** **..........** **..........** **.L........** **...R......** **..........** **.....K....** **..........**

**Kyonggi/SA1/2011**  **..........** **..........** **..........** **..........** **..........** **..........** **..........** **.....V....** **..........** **..........**

**Dubai/1/2012**  **..........** **..........** **..........** **..........** **..........** **.L........** **...R......** **..........** **.....K....** **..........**

**Rio Grande do Sul/1/2012** **..........** **..........** **..........** **..........** **..........** **.L........** **...R......** **..........** **.....K....** **..........**

**Tennessee/28B/2014**  **..........** **..........** **..........** **..........** **..........** **.L........** **..........** **..........** **..........** **..........**

**Montana/9564-1/2015**  **..........** **..........** **..........** **..........** **..........** **.L........** **..........** **..........** **..........** **..........**

**Malaysia/1/2015 ..........** **..........** **..........** **..........** **..........** **.L........** **..........** **..........** **..........** **..........**

**Georgia/121362-16/2016**  **..........** **..........** **..........** **..........** **..........** **.L........** **..........** **..........** **..........** **..........**

***110 120***

***....|....| ....|....| .***

**South Africa/4/2003**  **QALQLLLEVE** **QEIRTFSFQL** **I**

**Ohio/1/2003**  **..........** **.....**

**Ohio/113461-3/2005**  **..........** **..........** **.**

**Tottori/1/2007**  **..........** **..........** **.**

**Lincolnshire/1/2007**  **..........** **..........** **.**

**California/1/2010**  **..........** **..........** **.**

**Sweden/VIR165837/2011**  **..........** **..........** **M**

**Kentucky/1/2011**  **..........** **..........** **.**

**Kyonggi/SA1/2011**  **..........** **..........** **.**

**Dubai/1/2012**  **..........** **..........** **.**

**Rio Grande do Sul/1/2012** **..........** **..........** **.**

**Tennessee/28B/2014**  **..........** **..........** **.**

**Montana/9564-1/2015**  **..........** **..........** **.**

**Malaysia/1/2015**  **..........** **..........** **.**

**Georgia/121362-16/2016**  **..........** **..........** **.**
