# Supplementary material for: Whole Genome Sequencing of the First H3N8 Equine Influenza Virus Identified in Malaysia
Source: Pathogens. 2019 May 10;8(2):62. doi: 10.3390/pathogens8020062 (PMC6630255; doi:10.3390/pathogens8020062)
Supplement: Supplementary file 1 [file pathogens-08-00062-s001.zip › SupplementaryFiles/Supplementary Table S1.docx]

Supplementary Table S1: Accession codes for EIV HA1 and NA sequences included in phylogenetic analysis figures 1 and 2, respectively. GISAID accession numbers are highlighted in bold.

| **Year** | **Country** | **Isolate Name** | **HA1** | **NA** |
| --- | --- | --- | --- | --- |
|  |  |  | **accession** | **accession** |
| 1963 | USA | A/equine/Miami/1963 | M29257.1 | CY028838.1 |
| 1975 | USA | A/equine/New York/1/1975 | CY030189.1 | CY030191.1 |
| 1979 | France | A/equine/Fontainebleau/1979 | CY032405.1 | CY032407.1 |
| 1979 | United Kingdom | A/equine/Newmarket/1/1979 | KJ643908.1 | CY096893.1 |
| 1989 | Ireland | A/equine/Kildare/1989 | JN222941.1 | / |
| 1989 | USA | A/equine/Berlin/1/1989 | CY032413.1 | CY032415.1 |
| 1991 | USA | A/equine/Kentucky/1/1991 | CY030173.1 | CY030175.1 |
| 1992 | USA | A/equine/Kentucky/1/1992 | CY030149.1 | CY030151.1 |
| 1992 | Hong Kong | A/equine/Hong Kong/1/1992 | L27597.1 | / |
| 1992 | Ireland | A/equine/Kildare/1992 | JN084402.1 | / |
| 1993 | United Kingdom | A/equine/Newmarket/1/1993 | X85088.2 | FJ375222.1 |
| 1993 | United Kingdom | A/equine/Newmarket/2/1993 | X85089.2 | FJ375223.1 |
| 1994 | USA | A/equine/Kentucky/8/1994 | CY030181.1 | CY030183.1 |
| 1998 | USA | A/equine/Kentucky/1/1998 | AF197241.1 | / |
| 2003 | USA | A/equine/Ohio/1/2003 | DQ124192.1 | DQ124168.1 |
| 2003 | United Kingdom | A/equine/Newmarket/5/2003 | FJ375213.1 | FJ375224.1 |
| 2003 | South Africa | A/equine/South Africa/4/2003 | **EPI873601** | **EPI873600** |
| 2005 | Scotland | A/equine/Aboyne/1/2005 | EF541442.1 | KF049177.1 |
| 2005 | USA | A/equine/Ohio/113461-3/2005 | CY067326.1 | CY067328.1 |
| 2006 | United Kingdom | A/equine/Lincolnshire/1/2006 | FJ195399.1 | KF049176.1 |
| 2007 | Spain | A/equine/Spain/1/2007 | CY075851.1 | / |
| 2007 | USA | A/equine/New York/146066/2007 | CY067566.1 | / |
| 2007 | Switzerland | A/equine/Switzerland/P112/2007 | FJ195408.1 | **EPI584118** |
| 2007 | Ireland | A/equine/Meath/2007 | JN222935.1 | / |
| 2007 | United Kingdom | A/equine/Richmond/1/2007 | FJ195395.3 | KF559336.1 |
| 2007 | Japan | A/equine/Ibaraki/1/2007 | LC369072.1 | / |
| 2007 | Japan | A/equine/Tottori/1/2007 | AB591842.2 | AB591843.2 |
| 2007 | United Kingdom | A/equine/Lincolnshire/1/2007 | FJ195398.2 | KF559342.1 |
| 2008 | Ireland | A/equine/Down/1/2008 | JN222937.1 | / |
| 2009 | Japan | A/equine/Yokohama/aq19/2009 | AB544410.1 | / |
| 2009 | Ireland | A/equine/Donegal/1/2009 | JN222938.1 | / |
| 2009 | United Kingdom | A/equine/Yorkshire/3/2009 | **EPI584219** | **EPI584221** |
| 2009 | United Kingdom | A/equine/Dorset/1/2009 | CY054287.1 | KF049196.1 |
| 2009 | Ireland | A/equine/Carlow/1/2009 | JN222939.1 | / |
| 2010 | Ireland | A/equine/Limerick/1/2010 | JN222940.1 | / |
| 2010 | Ireland | A/equine/Kildare/2/2010 | KC871537.1 | / |
| 2010 | France | A/equine/Eyragues/1/2010 | JX091785.1 | / |
| 2010 | USA | A/equine/California/1/2010 | **EPI584270** | **EPI584272** |
| 2011 | Sweden | A/equine/Sweden/VIR165837/2011 | **EPI594013** | **EPI594018** |
| 2011 | South Korea | A/equine/Kyonggi/SA1/2011 | JX844146.2 | JX844148.2 |
| 2011 | Ireland | A/equine/Kilkenny/2011 | KC871545.1 | / |
| 2011 | Ireland | A/equine/Carlow/2011 | KC871546.1 | / |
| 2011 | Mongolia | A/equine/Mongolia/3/2011 | JX549062.1 | / |
| 2011 | USA | A/equine/Pennsylvania/6-15/2011 | KF026406.1 | KF049179.1 |
| 2011 | USA | A/equine/Kentucky/1/2011 | **EPI584278** | **EPI584280** |
| 2012 | United Kingdom | A/equine/County Durham/2/2012 | KF026396.1 | KF049192.1 |
| 2012 | Germany | A/equine/Lichtenfeld/1/2012 | JX499136.1 | KF049191.1 |
| 2012 | Ireland | A/equine/Kildare/2/2012 | **EPI526643** | / |
| 2012 | United Arab Emirates | A/equine/Dubai/1/2012 | KF026411.1 | KF049173.1 |
| 2012 | Germany | A/equine/Rastatt/1/2012 | KC584975.1 | / |
| 2012 | USA | A/equine/Kentucky/2/2012 | KF026408.1 | KF049187.1 |
| 2012 | Kazakhstan | A/equine/South Kazakhstan/236/2012 | KF712451.1 | KF712452.1 |
| 2012 | Brazil | A/equine/Rio Grande do Sul/1/2012 | **EPI584295** | **EPI584297** |
| 2012 | USA | A/equine/Kentucky/3/2012 | KF026409.1 | KF049186.1 |
| 2013 | China | A/equine/Heilongjiang/SS1/2013 | KC986390.2 | KC986392.2 |
| 2013 | United Kingdom | A/equine/Lanarkshire/1/2013 | **EPI493612** | **EPI493613** |
| 2013 | Turkey | A/equine/Ankara/1/2013 | MF067527.1 | MF067529.1 |
| 2014 | USA | A/equine/Tennessee/28B/2014 | KR351241.1 | KR351243.1 |
| 2014 | Germany | A/equine/North Rhine Westphalia/1/2014 | KJ538149.1 | / |
| 2014 | United Kingdom | A/equine/Buckinghamshire/1/2014 | **EPI651398** | **EPI651400** |
| 2014 | France | A/equine/Gironde/1/2014 | KY241319.1 | KY241352.1 |
| 2015 | Brazil | A/equine/Sao Paulo/32.FMVZ/2015 | KX954143.1 | / |
| 2015 | USA | A/equine/Montana/9564-1/2015 | MG198999.1 | MG199001.1 |
| 2015 | United Kingdom | A/equine/Kent/1/2015 | **EPI873593** | **EPI873592** |
| 2015 | France | A/equine/Saone-et-Loire/1/2015 | KY241320.1 | KY241353.1 |
| 2016 | United Kingdom | A/equine/Kent/1/2016 | **EPI957608** | **EPI957609** |
| 2016 | USA | A/equine/Georgia/121362-16/2016 | MF173124.1 | MF173198.1 |
| 2017 | Japan | A/equine/Yokohama/aq100/2017 | LC269107.1 | / |
