# Supplementary material for: Whole Genome Sequencing of the First H3N8 Equine Influenza Virus Identified in Malaysia
Source: Pathogens. 2019 May 10;8(2):62. doi: 10.3390/pathogens8020062 (PMC6630255; doi:10.3390/pathogens8020062)
Supplement: Supplementary file 1 [file pathogens-08-00062-s001.zip › SupplementaryFiles/Supplementary Table S2.docx]

Supplementary Table S2: Accession codes for sequences used in figure 3 and supplementary alignments. GISAID accession numbers are highlighted in bold.

|  | | | **Segment and Segment Accession** | | | | | | | |
| --- | --- | --- | --- | --- | --- | --- | --- | --- | --- | --- |
| **Source** | **Country** | **Isolate Name** | **PB2** | **PB1** | **PA/PA-X** | **HA** | **NP** | **NA** | **MP** | **NS** |
| NCBI | USA | A/eq/Ohio/1/2003 | DQ124177 | DQ124172 | DQ124187.2 | DQ124192 | DQ124184 | DQ124168 | DQ124188 | DQ124186 |
| NCBI | USA | A/eq/Ohio/113461-3/2005 | CY067323 | CY067324 | CY067325 | CY067326 | CY067327 | CY067328 | CY067329 | CY067330 |
| GISAID | UK | A/eq/Lincolnshire/1/2007 | KF559338 | KF559339 | KF559340 | FJ195398 | KF559341 | KF559342 | KF559343 | FJ195427 |
| NCBI | Japan | A/eq/Tottori/1/2007 | AB591847 | AB591846 | AB591845 | AB591842 | AB591844 | AB591843 | AB591848 | AB591849 |
| NCBI | South Korea | A/eq/Kyonggi/SA1/2011 | JX844143.2 | JX844144.2 | JX844145.2 | JX844146.2 | JX844147.2 | JX844148.2 | JX844149.2 | JX844150.2 |
| NCBI | USA | A/eq/Tennessee/28B/2014 | KR351238 | KR351239 | KR351240 | KR351241 | KR351242 | KR351243 | KR351244 | KR351245 |
| NCBI | USA | A/eq/Montana/9564-1/2015 | MG198996 | MG198997 | MG198998 | MG198999 | MG199000 | MG199001 | MG199002 | MG199003 |
| NCBI | USA | A/eq/Georgia/121362-16/2016 | MF173384 | MF173380 | MF173264 | MF173124 | MF173210 | MF173198 | MF173141 | MF173137 |
| GISAID | South Africa | A/eq/South Africa/4/2003 | **EPI873602** | N/A | **EPI873603** | **EPI873601** | **EPI873605** | **EPI873600** | **EPI873606** | **EPI873604** |
| GISAID | USA | A/eq/California/1/2010 | **EPI584266** | **EPI584267** | **EPI584268** | **EPI584270** | **EPI584271** | **EPI584272** | **EPI584273** | **EPI584274** |
| GISAID | Sweden | A/eq/Sweden/VIR165837/2011 | **EPI594130** | **EPI594111** | **EPI594054** | **EPI594013** | **EPI594033** | **EPI594018** | **EPI594010** | **EPI594042** |
| GISAID | USA | A/eq/Kentucky/1/2011 | **EPI584275** | **EPI584276** | **EPI584277** | **EPI584278** | **EPI584279** | **EPI584280** | **EPI584281** | **EPI584282** |
| GISAID | United Arab Emirates | A/eq/Dubai/1/2012 | **EPI584283** | **EPI584284** | **EPI584285** | **EPI584286** | **EPI584287** | **EPI584288** | **EPI584289** | **EPI584290** |
| GISAID | Brazil | A/eq/Rio Grande Do Sol/1/2012 | **EPI584292** | **EPI584293** | **EPI584294** | **EPI584295** | **EPI584296** | **EPI584297** | **EPI584298** | **EPI584299** |
